# Metabolic Profile of the Genome-Reduced *Bacillus subtilis* Strain IIG-Bs-27-39: An Attractive Chassis for Recombinant Protein Production

**DOI:** 10.1021/acssynbio.4c00254

**Published:** 2024-07-09

**Authors:** Rocío Aguilar Suárez, Michael Kohlstedt, Ayşegül Öktem, Jolanda Neef, Yuzheng Wu, Kaiya Ikeda, Ken-Ichi Yoshida, Josef Altenbuchner, Christoph Wittmann, Jan Maarten van Dijl

**Affiliations:** † Department of Medical Microbiology, University Medical Center Groningen-University of Groningen, 9700RB Groningen, The Netherlands; ‡ Institute for Systems Biotechnology, Saarland University, 66123 Saarbrücken, Germany; § Department of Science, Technology and Innovation, Kobe University, 657-8501 Kobe, Japan; ∥ Institute for Industrial Genetics, University of Stuttgart, 70569 Stuttgart, Germany

**Keywords:** *Bacillus subtilis*, genome reduction, metabolic features, energetic parameters

## Abstract

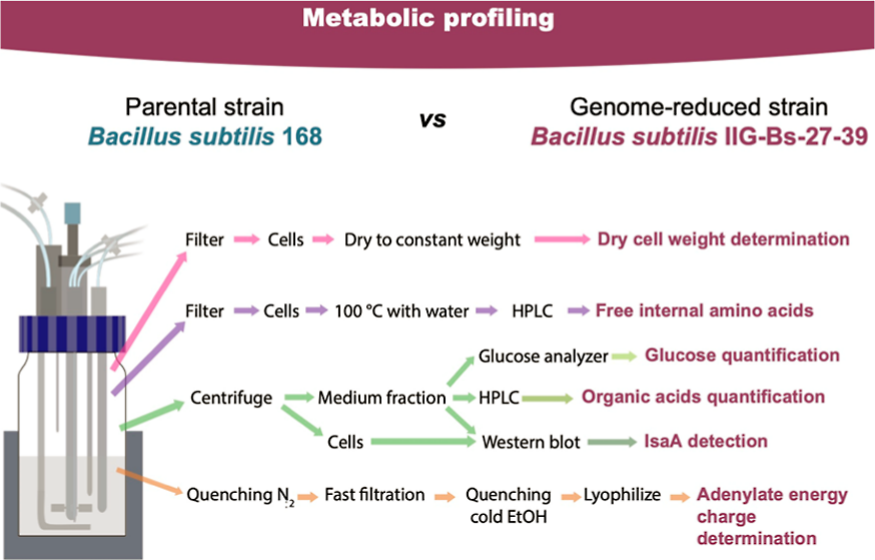

The Gram-positive bacterium *Bacillus subtilis* is extensively used in the industry for the secretory production of proteins with commercial value. To further improve its performance, this microbe has been the subject of extensive genome engineering efforts, especially the removal of large genomic regions that are dispensable or even counterproductive. Here, we present the genome-reduced *B. subtilis* strain IIG-Bs-27-39, which was obtained through systematic deletion of mobile genetic elements, as well as genes for extracellular proteases, sporulation, flagella formation, and antibiotic production. Different from previously characterized genome-reduced *B. subtilis* strains, the IIG-Bs-27-39 strain was still able to grow on minimal media. We used this feature to benchmark strain IIG-Bs-27-39 against its parental strain 168 with respect to heterologous protein production and metabolic parameters during bioreactor cultivation. The IIG-Bs-27-39 strain presented superior secretion of difficult-to-produce staphylococcal antigens, as well as higher specific growth rates and biomass yields. At the metabolic level, changes in byproduct formation and internal amino acid pools were observed, whereas energetic parameters such as the ATP yield, ATP/ADP levels, and adenylate energy charge were comparable between the two strains. Intriguingly, we observed a significant increase in the total cellular NADPH level during all tested conditions and increases in the NAD^+^ and NADP(H) pools during protein production. This indicates that the IIG-Bs-27-39 strain has more energy available for anabolic processes and protein production, thereby providing a link between strain physiology and production performance. On this basis, we conclude that the genome-reduced strain IIG-Bs-27-39 represents an attractive chassis for future biotechnological applications.

## Introduction

Bacterial cell factories are in the limelight of biotechnological applications to provide an extended spectrum of products including enzymes, amino acids, organic acids, steroids, vitamins, and antibiotics.^1,2^ The exploitation of these living cells to synthesize such commodities is expected to expand even further in order to meet the ever growing societal demands for more sustainable and environmentally friendly processes to replace polluting chemical processes, to tap new natural resources, or to deliver novel medicines.^3−5^

Cells used in industrial biotechnology have been randomly mutagenized and modified genetically with the aim to improve their production capabilities. Nowadays, new strains with engineered traits can be rationally designed and created through synthetic biological approaches, including the refactoring of complete genomes.^6,7^ One of the entertained approaches involves genome minimization, which is highly feasible for bacteria. For instance, bacteria that have been subject to extensive genome reduction include *Pseudomonas putida*, *Corynebacterium glutamicum*, *Escherichia coli,* and, last but not least, *Bacillus subtilis*.^8−13^ Nonetheless, only a few genome-reduced derivative strains have been tested for production of value-added compounds.^6,13−20^

*B. subtilis* is an attractive host for protein production due to its high secretion capacity, allowing yields of more than 25 g/L of secreted protein, and the “Generally Recognized As Safe” status, given to many of its products by the Food and Drug Safety Authority of the United States.^21^*B. subtilis*, as a natural inhabitant of the soil and plant rhizosphere, has an extensive gene repertoire that allows its adaptation to extreme environmental conditions.^22^ However, the high genetic versatility of *B. subtilis* may not be necessary in industrial settings, where culture conditions are highly controlled. Moreover, the expression of nonessential genes during industrial fermentation could potentially result in a waste of energy and cellular resources that should preferably be redirected toward product formation. For instance, the synthesis of flagella and fimbriae requires carbon and energy resources, but these cellular appendices are not needed in a stirred bioreactor.^23^ Furthermore, *B. subtilis* may not perform optimally even under ideal conditions since part of its resources are used to maintain an “alert” state to prepare the bacteria for sudden changes in environmental conditions.^24,25^ Thus, the removal of nonessential genes and gene clusters could lead to more efficient cell factories.^26^ Moreover, genome reduction is supposed to decrease the complexity of cells and reduce heterogeneity in bacterial populations, especially through the removal of mobile genetic elements.^7,27^ Hence, genome reduction has emerged as a useful tool, not only to elucidate the function of genetic elements but also to design completely new strains for biotechnological applications.^28−30^

Genome-reduced derivatives of *B. subtilis* strain 168, including the mini*Bacillus* strain PG10 and the midi*Bacillus* strain IIG-Bs-27-47-24, were recently shown to have beneficial traits for protein production, including an increased capacity for translation and protein secretion combined with the absence of the main extracellular proteases.^6,31^ However, these strains lost the ability to grow in particular minimal media. The latter deficiency precluded studies on the metabolic features of such genome-reduced strains and represented a drawback for their use in production scenarios based on minimal nutrient formulations. Since we considered it important to understand the metabolic adaptations of such strains for further strain engineering, the present study was aimed at identifying a strain from the genome engineering phylogeny that combined beneficial traits in terms of protein production with growth on minimal media. In brief, this search led to the identification of *B. subtilis* strain IIG-Bs27-39, which lacks 26.1% of the genome compared to the 168 strain. Genes that are absent from the IIG-Bs-27-39 strain encoded for proteins involved in extracellular proteolysis, chemotaxis, mobility, antibiotic production, biofilm formation, and sporulation. Next, we explored the growth of strain IIG-Bs-27-39 in bioreactors and assessed its ability to produce a heterologous secretory model protein, the immunodominant staphylococcal antigen IsaA. IsaA was selected for our study because it was previously shown that it is difficult to produce and barely secreted in *B. subtilis* 168.^6^ Importantly, there is biomedical interest in IsaA because it may serve as an antigen for vaccination against infections by the multidrug-resistant human and animal pathogen *Staphylococcus aureus*.^32−34^ Our present benchmarking of the IIG-Bs-27-39 strain against the parental strain 168 shows the superiority of the IIG-Bs-27-39 strain with respect to the specific growth rate, biomass yield coefficient, and protein production.

## Results

### Selection of the Genome-Reduced Strain IIG-Bs-27-39 for Protein Production

**Figure 1:**
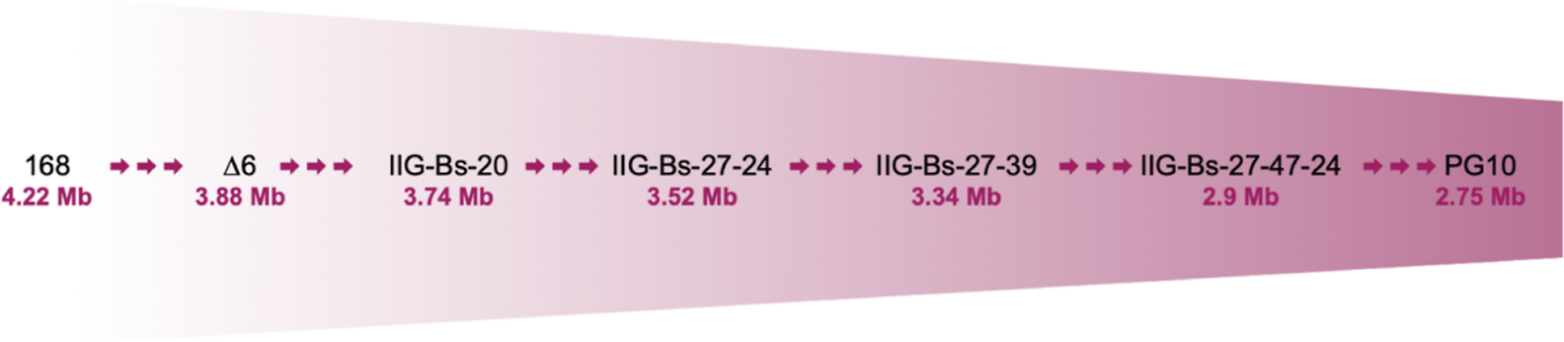
Phylogeny of genome-reduced *B. subtilis* strains. Strain names and the genome sizes of important intermediate strains are indicated. Multiple arrows indicate several steps of genome reduction.

**Figure 2:**
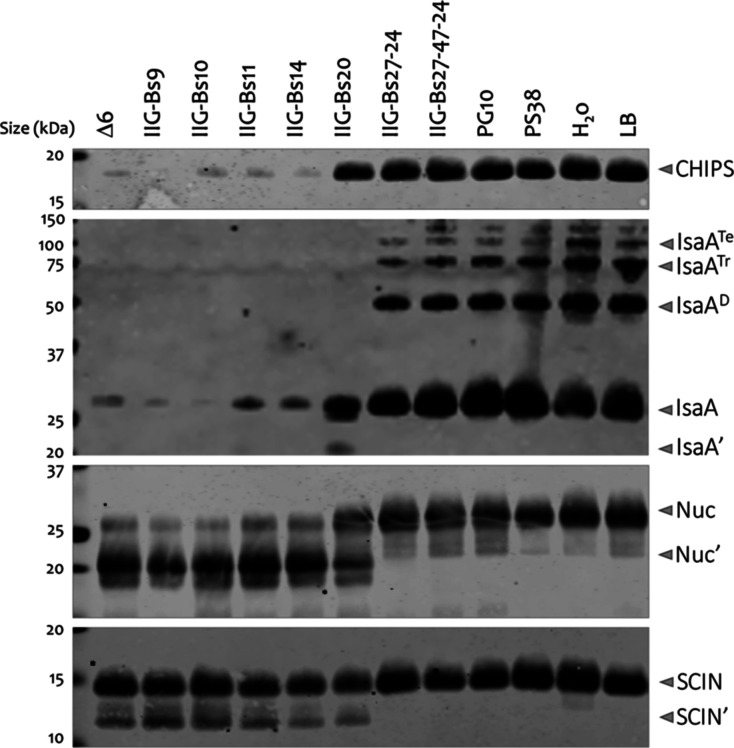
Stability of staphylococcal proteins in spent media of *B. subtilis* genome-reduced strains. Culture supernatants from *L. lactis* overexpressing the staphylococcal proteins CHIPS, IsaA, Nuc, or SCIN were mixed with cell-free culture supernatants of genome-engineered *B. subtilis* strains grown in LB (for details on strains see Table 1). Proteins were incubated for 2 h at 37 °C and then TCA-precipitated to assess their degradation by western blotting. The CHIPS and Nuc proteins were detected with anti-his_6_ antibodies, SCIN was detected with the human monoclonal antibody 6D4, and IsaA with the human monoclonal antibody 1D9. IsaA′, IsaA degradation product; IsaA^D^, IsaA dimer; IsaA^Tr^, IsaA trimer; IsaA^Te^, IsaA tetramer; Nuc′, Nuc degradation products; and SCIN′, SCIN degradation product.

First, we screened for a strain from the *B. subtilis* 168 genome minimization phylogeny that combined maximal heterologous protein stability with proficient growth on minimal media (Figure 1). This involved production of a panel of reporter proteins from *S. aureus* in *Lactococcus lactis* and their subsequent exposure to spent growth media of genome-minimized strains. This revealed a marked reduction of reporter protein degradation in the spent medium of strain IIG-Bs-27-24, whereas most reporter proteins were extensively degraded in the spent medium of strain IIG-Bs-20, which is positioned “upstream” in the phylogeny of genome-minimized strains (Figure 2). Notably, from strain IIG-Bs-27-31 onward, all genome-minimized strains lacked the genes for the eight major extracellular proteases of *B. subtilis*, which is a property that we wanted to maintain. Therefore, we inspected strains downstream of strain IIG-Bs-27-31 for growth on different media using shake flasks. This showed that genome-reduced strains up until strain IIG-Bs-27-47 were able to grow in the MBU and PMM media (data not shown). From the genome-reduced strains downstream of strain IIG-Bs-27-31, we selected strain IIG-Bs-27-39 for further analysis because it lacks various genes involved in sporulation and spore germination and is able to grow well in the M9 minimal medium that is appropriate for metabolic analyses. The IIG-Bs-27-39 strain lacks ∼21.6% of the genome of the parental strain 168, and the deleted genomic regions are shown in Table S1.

### Enhanced Secretion of IsaA in M9 Medium by Strain IIG-Bs-27-39

To verify effective production of the immunodominant staphylococcal antigen IsaA of *S. aureus* by the IIG-Bs-27-39 strain, we made use of the previously developed subtilin-inducible SURE expression system.^35^ To this end, the *spaRK* genes were inserted in the *amyE* locus, and the plasmid pRAG3::*isaA* was introduced in the IIG-Bs-27-39 strain. In the resulting strain, the SpaK histidine kinase will sense the presence of subtilin added to the growth medium, resulting in phosphorylation of the SpaR response regulator which, in turn, will lead to induced expression of the *isaA* gene from the *spaS* promoter on pRAG3::*isaA*.^6^

**Figure 3:**
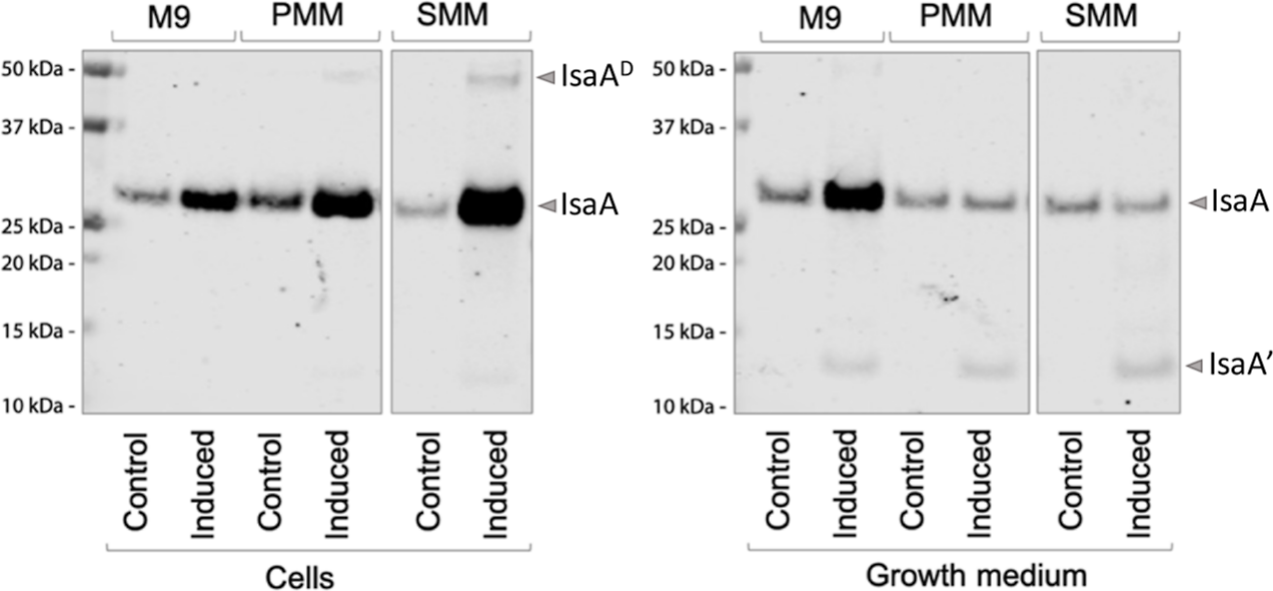
IsaA production and secretion by strain IIG-Bs-27-39 cultivated in different minimal media. The IIG-Bs-27-39 cells carrying the *spaRK* genes and pRAG3::*isaA* were grown in M9, SMM, or PMM minimal media and induced with 1% subtilin during the exponential growth phase. Culture samples were collected after 5 h of incubation post induction and corrected for an OD_600_ value of 2.0 before protein separation by LDS-PAGE. The production and secretion of IsaA were visualized by western blotting with the IsaA-specific monoclonal antibody 1D9. IsaA^D^, dimer of IsaA; IsaA′, degradation product of IsaA.

The first step toward characterization of the IIG-Bs-27-39 strain as a potential cell factory for production of the IsaA reporter protein was the selection of an appropriate minimal medium for cultivation. Therefore, we evaluated IsaA production in shake-flask cultures with established M9, SMM, or PMM minimal media. The main difference between these three media is the presence of citrate in PMM and SMM, whereas citrate is absent from the M9 medium. Cells were grown to the exponential phase, and, at this point, IsaA production was induced with 1% subtilin. The cultures were further incubated for 5 h and, subsequently, IsaA production and secretion were assessed by western blotting. The results pointed out differences in the production of IsaA by the IIG-Bs-27-39 strain depending on the growth medium (Figure 3). The amount of IsaA produced and accumulated in the cell fraction was higher when the bacteria were grown in SMM or PMM. However, when the IIG-Bs-27-39 strain was cultivated in M9, IsaA was secreted at much higher levels than upon cultivation in SMM or PMM. Therefore, M9 medium was selected for the cultivation of the IIG-Bs-27-39 strain in bioreactors. An additional advantage of M9 was that, in this medium, glucose is the only carbon source present. To note, we also detected IsaA expression in the noninduced cells, indicating a less strict repression of the inducible *spaS* promoter in the IIG-Bs-27-39 strain than was previously observed for the midi*Bacillus* strain IIG-Bs-27-47-24.^36^

### Metabolic Features of the Genome-Reduced IIG-Bs-27-39 Strain

**Figure 4:**
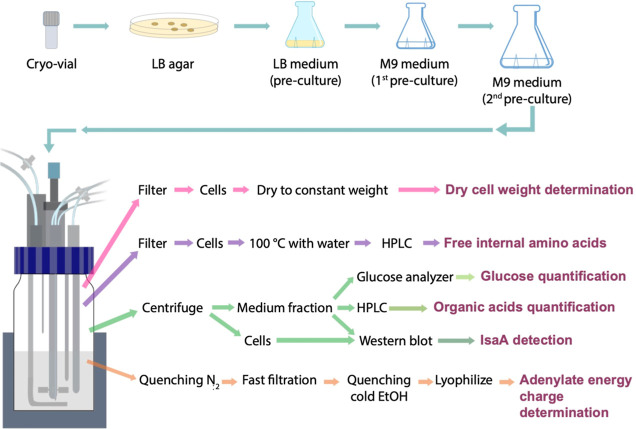
Overview of the experimental workflow to analyze samples collected from the bioreactors. Single colonies from the parental strain 168 or the genome-reduced strain IIG-Bs-27-39, both carrying the *spaRK* genes and plasmid pRAG3::*isaA*, were used to inoculate initial precultures in the LB medium, which was followed by two preculture steps in the M9 minimal medium. Cells from the last preculture were used to inoculate 1 L bioreactors to an initial OD_600_ value of 0.1 in 300 mL of M9 minimal medium. Production of the staphylococcal protein IsaA was induced with 1% subtilin when the cultures reached an OD_600_ value of ∼1. Aliquots were collected from the bioreactors at different time points for further analyses. The workflow summarizes the main steps in sample processing and analysis.

To evaluate the performance of the genome-reduced IIG-Bs-27-39 strain, the IIG-Bs-27-39 strain and the parental strain 168, both carrying the *spaRK* genes and plasmid pRAG3::*isaA*, were cultured in 1 L lab-scale bioreactors under defined conditions with glucose as the only carbon source. An overview of the experimental workflow is described in Figure 4. We hypothesized that there might be differences in the performance of both strains due to the genome reduction in the IIG-Bs-27-39 strain. To this end, we analyzed several physiological parameters related to growth and metabolite secretion such as the biomass yield coefficient (*Y*_X/S_), maximum specific growth rate (μ_max_), substrate uptake rate (*q*_S_), acetate yield (*Y*_Ace/S_), specific acetate secretion rate (*q*_Ace_), pyruvate yield (*Y*_Pyr/S_), and specific pyruvate secretion rate (*q*_Pyr_) (Table 2). Indeed, during cultivation, strain IIG-Bs-27-39 exhibited a much higher biomass yield coefficient compared to the parental strain. Similarly, the maximum specific growth rate measured for strain IIG-Bs-27-39 was 20% higher than that of the parental strain. Although the IIG-Bs-27-39 strain produced more biomass than the 168 strain, it lost more carbon through the formation of acetate and CO_2_ (Table 2 and Figure S2). Instead, the parental strain 168 showed a higher yield of pyruvate (Table 2).

**Table 1:** Strains and Plasmids Used in This Study^a^

strain	characteristics	reference
*Bacillus subtilis*		
168	parental strain, *trpC2*	(57)
168 *spaRK*	derivative of the parental *B. subtilis* 168 strain, *trp*^+^, *amyE*::*spaRK*-Km^R^. The Km^R^ marker was introduced in the *amyE* gene together with the *spaRK* genes	this study
Δ6	*trpC2* Δ*SP*β, Δ*skin*, Δ*PBSX*, Δ*pro*ϕ*1*, Δ*pks*::*cat*, Δ*pro*ϕ*3*; *Cm*^R^	(58)
IIG-Bs-9	genome-reduced derivative of strain 168 that is parental to IIG-Bs-10. See Table S1 for detailed genotype	(59)
IIG-Bs-10	genome-reduced derivative of strain 168 that is parental to IIG-Bs-11. See Table S1 for detailed genotype	(59)
IIG-Bs-11	genome-reduced derivative of strain 168 that is parental to IIG-Bs-14. See Table S1 for detailed genotype	(59)
IIG-Bs-14	genome-reduced derivative of strain 168 that is parental to IIG-Bs-20. See Table S1 for detailed genotype	(59)
IIG-Bs-20	genome-reduced derivative of strain 168 that is parental to IIG-Bs-27-7. See Table S1 for detailed genotype	(59)
IIG-Bs-27-7	Genome-reduced derivative of strain 168 that is parental to IIG-Bs-27-24. See Table S1 for detailed genotype	(60)
IIG-Bs-27-24	genome-reduced derivative of strain 168 that is parental to IIG-Bs-27-39. See Table S1 for detailed genotype	(43)
IIG-Bs-27-39	genome-reduced derivative of strain 168 that is parental to IIG-Bs-27-47-24. See Table S1 for detailed genotype	(60)
IIG-Bs-27-39 *spaRK*	IIG-Bs-27-39 derivative, *amyE*::*spaRK*, Km^R^	this study
IIG-Bs-27-47-24	genome-reduced derivative of strain 168 that is parental to IIG-Bs-PG10. See Table S1 for detailed genotype	(43)
PG10	genome-reduced derivative of strain 168. See Table S1 for detailed genotype	(43)
PS38	genome-reduced derivative of strain 168. See Table S1 for detailed genotype	(43)
ATCC 6633	subtilin producer	ATCC collection
*Escherichia coli*		
JM109	cloning host for the construction of *B. subtilis* strain IIG-Bs27-39	(61)
DH5-α	cloning host for the construction of pBSMul1-nuc-11	
*Lactococcus lactis*		
PA1001	MG1363 *pepN*::*nisRK*, allows nisin-inducible expression, Δ*acmA* Δ*htrA*	mucosis culture collection, Groningen, The Netherlands
NZ9700	nisin producer	(62)
Plasmids		
pRAG3::*isaA*	Em^R^, subtilin-regulated SP_*xynA*_-*isaA* expression	(6)
pNZ8910	carries the *spaRK*-Km^R^ module with the flanking regions of the *amyE* gene for double crossover insertion into the *amyE* locus	(35)
pJOE6743.1	Sp^R^ (selection marker) and P*man*P-*man*P (antiselection marker)	(59)
pBSMul1-cut-11	Km^R^, Amp^R^, strong constitutive promoter for Gram-positive bacteria (P*Hpa*I*I*)	(63)
pBSMul1-nuc-11	Km^R^, constitutive expression of the staphylococcal thermonuclease Nuc	this study
pRAG3::*nuc*	encodes Nuc fused to the signal peptide of XynA	(6)
pNG4210::*chp*	pNG4210 encoding CHIPS with a C-terminal his_6_	(64)
pNG4210::*SCIN*	pNG4210 encoding SCIN with C-terminal his_6_	(64)
pNG4210::*isaA*	pNG4210 encoding IsaA with C-terminal his_6_	laboratory collection
pNG400::*nuc*	pNG400 encoding Nuc with C-terminal his_6_	(65)

a Km^R^: kanamycin resistance. Amp^R^: ampicillin resistance. Em^R^: erythromycin resistance. Sp^R^: spectinomycin resistance. Cm^R^: chloramphenicol resistance.

**Table 2:** Physiological Parameters in Bioreactor Cultures of Strain IIG-Bs-27-39 and the Parental Strain 168^a^

parameter	strain	mean	SD^b^	units
*Y*_X/S_	parental strain	44.66	1.65	g_DCW_/mol
	IIG-Bs-27-39	74.48	1.04	
μ_max_	parental strain	0.43	0.01	1/h
	IIG-Bs-27-39	0.52	0.01	
*q*_S_	parental strain	9.70	0.42	mmol/g_DCW_/h
	IIG-Bs-27-39	7.02	0.15	
*Y*_Ace/S_	parental strain	306.20	19.13	mmol/mol
	IIG-Bs-27-39	561.99	79.43	
*q*_Ace_	parental strain	2.97	0.23	mmol/g_DCW_/h
	IIG-Bs-27-39	3.95	0.56	
*Y*_Pyr/S_	parental strain	14.17	1.18	mmol/mol
	IIG-Bs-27-39	6.68	0.22	
*q*_Pyr_	parental strain	0.14	0.02	mmol/g_DCW_/h
	IIG-Bs-27-39	0.05	0.01	

a Strain IIG-Bs-27-39 and the parental strain 168, both carrying the *spaRK* genes and pRAG3::*isaA*, were grown in batch cultures in M9 minimal medium with 5 g/L glucose as sole carbon source. Subtilin was added to induce IsaA production.

b Results represent the mean value of the parameter and the standard deviation (SD) for measurements in two independent biological replicate experiments.

**Figure 5:**
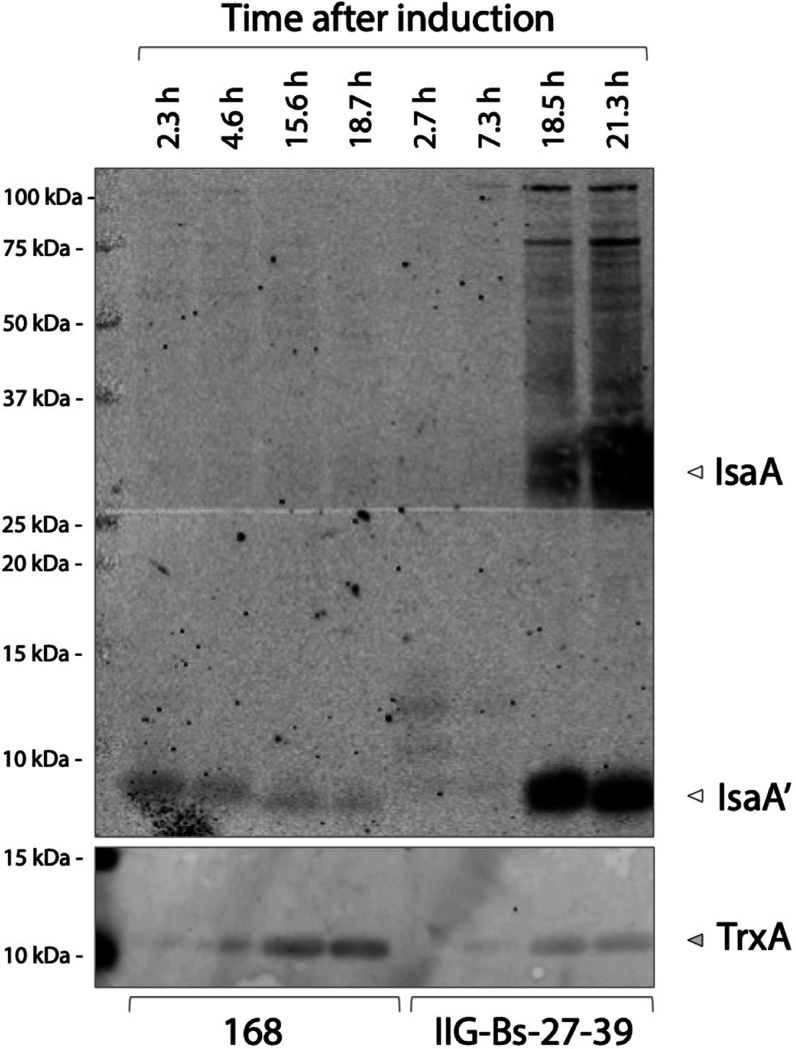
Secretion of the model protein IsaA by strain IIG-Bs-27-39 and the parental strain 168 in bioreactor batch cultures. Medium samples of strains IIG-Bs-27-39 and 168, both carrying the *spaRK* genes and pRAG3::*isaA*, were collected at different time points after subtilin-induced IsaA production. Samples were corrected to an OD_600_ value of 2 before LDS-PAGE and western blotting with the IsaA-specific human monoclonal antibody 1D9. Please note that, since the production of IsaA was induced at equal OD_600_ values for both strains, the induction time points were not identical. Together with the different metabolome analyses that had to be carried out and the fact that four fermentations were run in parallel, this caused slight shifts in the time points of sampling. IsaA′ refers to a major degradation product of IsaA.

At different time points after subtilin induction, we collected samples to analyze the secretion of IsaA by western blotting. IsaA was detected in the growth medium of the parental strain, but only in the form of degradation products with a lower molecular weight than that of the full-size IsaA (Figure 5). On the other hand, the genome-reduced strain IIG-Bs-27-39 secreted full-size mature IsaA into the growth medium, which was detected both in the form of monomers and oligomers. Nonetheless, degradation fragments of IsaA were also detected, especially at late time points after induction when a major degradation product was detectable (Figure 5; protein-labeled IsaA′). The improved accumulation of full-size IsaA in the medium of the IIG-Bs-27-39 strain can be attributed to the successive deletion of protease genes during the construction of this strain (Table S1). However, it is noteworthy that the growth medium of strain IIG-Bs-27-39 still displayed an IsaA-degrading activity of unknown origin. Another important finding is that the IIG-Bs-27-39 strain appears to be more resistant to cell lysis, as shown by relatively low levels of the cytoplasmic marker protein thioredoxin A (TrxA) as compared to the 168 strain (Figure 5).

### Amino Acid Pools, Overflow Metabolism, and Energetic Levels

**Figure 6:**
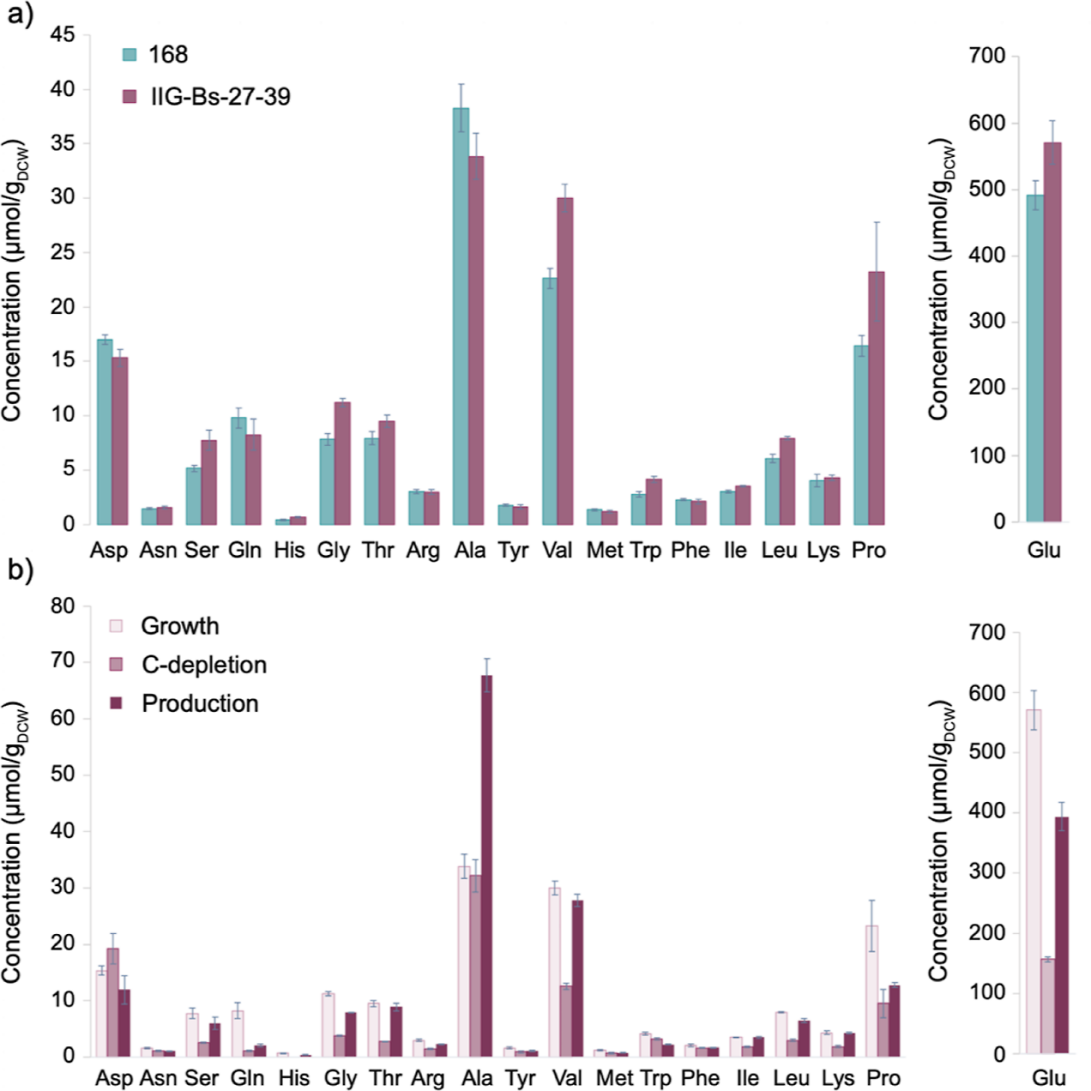
Intracellular pools of amino acids during batch cultivation in bioreactors. Cell cultures were harvested *via* vacuum filtration. Amino acids were extracted from the cells and quantified *via* HPLC. (a) Amino acid pools in exponentially growing bacteria of strain IIG-Bs-27-39 and the parental strain 168, both carrying the *spaRK* genes and pRAG3::*isaA*. (b) Comparison of amino acid pools in strain IIG-Bs-27-39 *spaRK* carrying pRAG3::*isaA* during exponential growth, upon carbon depletion and in the production phase. The data represent the mean values and standard deviation of two biological replicates.

To exclude possible limitations related to the abundance of amino acids, we measured the internal amino acid pools in the IIG-Bs-27-39 and 168 strains after induction of IsaA production. First, we compared the amino acid pools during the exponential growth phase of both strains. Here, we observed a slightly higher abundance of serine, glycine, valine, and leucine in the IIG-Bs-27-39 strain, while the aspartate and alanine abundance were slightly lower than in the parental strain (Figure 6a). Interestingly, we observed that the amount of alanine increased by one-fold during the production phase (Figure 6b), despite the fact that alanine is the most abundant amino acid in the IsaA protein, followed by serine and glycine. Moreover, we also observed a recovery in the amino acid pools of 13 of the 19 quantified amino acids during the production phase in strain IIG-Bs-27-39 (Figure 6b).

**Figure 7:**
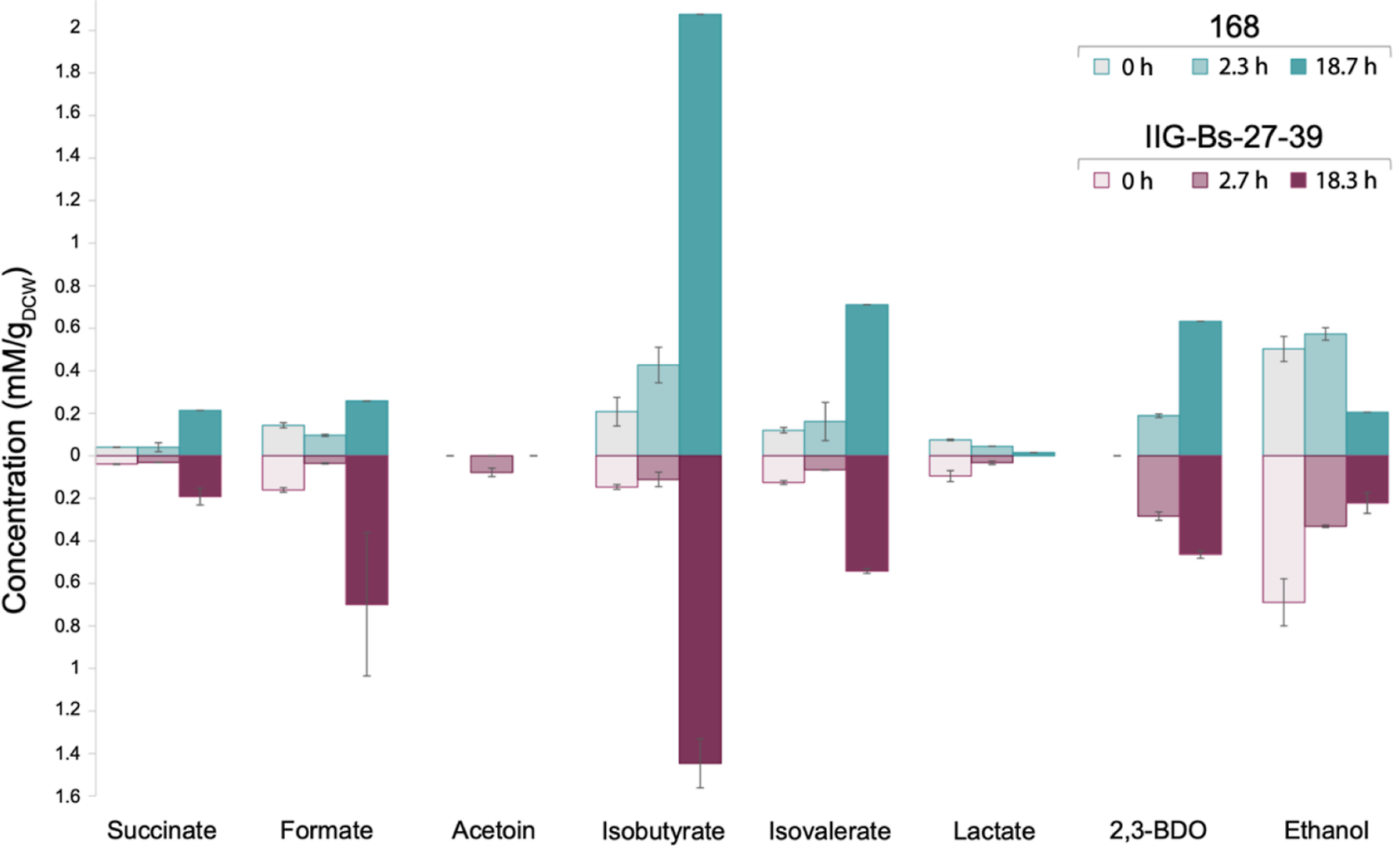
Secretion of organic acids and byproducts during batch cultivation in bioreactors. The concentration of the compounds in the medium fraction of strain IIG-Bs-27-39 and the parental strain 168, both carrying the *spaRK* genes and pRAG3::*isaA*, was measured at different time points after the induction of IsaA production. The concentrations measured for the parental strain are indicated in the upper part of the plot, and the concentrations measured for strain IIG-Bs-27-39 in the lower part. The data represent the mean values and standard deviation in the measurements for two biological replicates.

A relevant feature in industrial settings is the efficient utilization of carbon sources for product formation. An excess of carbon in a culture can result in a higher rate of carbon consumption but also create an imbalance between anabolic and catabolic reactions. In this state, cells direct their resources toward overflow metabolism, which leads to a less efficient use of carbon and energy resources.^37^ Therefore, we investigated the levels of key secreted overflow metabolites and byproducts, such as ethanol, acetate, acetoin, succinate, formate, isobutyrate, isovalerate, 2,3-butanediol, and lactate, in the parental strain 168 and the IIG-Bs-27-39 strain. Differences were observed regarding the synthesis of isobutyrate, isovalerate, and 2,3-butanediol after ∼18 h of induction, where the parental strain excreted higher amounts compared to the IIG-Bs-27-39 strain (Figure 7). Instead, the higher growth rate and biomass formation by the IIG-Bs-27-39 strain correlated with higher production of acetoin and acetate (Figure 7 and Table 2).

**Figure 8:**
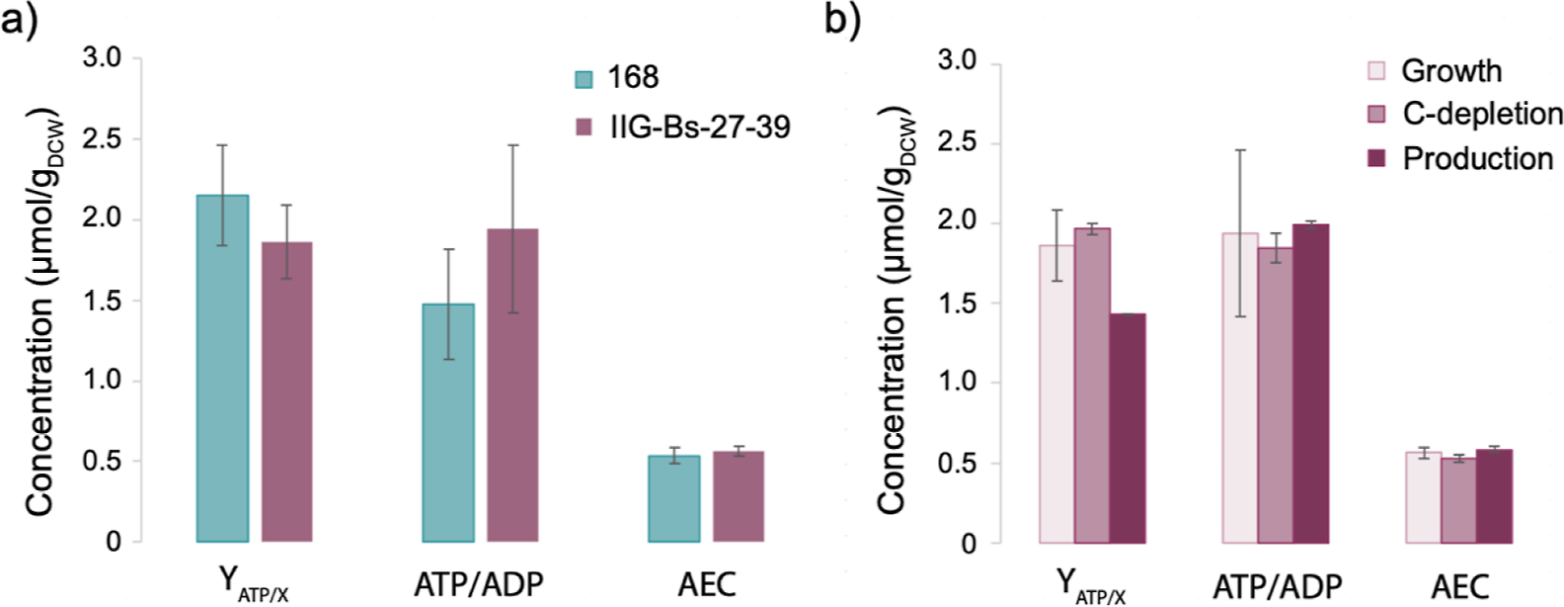
Energy charge of the parental strain 168 and strain IIG-Bs-27-39 during batch cultivation in bioreactors. The ATP yields, ATP/ADP levels, and AEC were measured for (a) parental strain and the IIG-Bs-27-39 strain during exponential growth and (b) for strain IIG-Bs-27-39 at different culture conditions. Both strains carried the *spaRK* genes and pRAG3::*isaA*. The data represent the mean values and standard deviation in the measurements for two biological replicates. Note that the AEC values are “unit-less”.

To evaluate how the genome reduction in strain IIG-Bs-27-39 affected the cellular energy levels, we measured the AMP, ADP, and ATP content of the cells. Particularly, the ATP/ADP ratio and the adenylate energy charge (AEC) were used to estimate the energy capacity of the strains.^38^ We hypothesized that the deletion of nonessential genes could reduce the waste of energy resources in strain IIG-Bs-27-39, but this strain’s ATP yield, ATP/ADP levels, and AEC were comparable to those of the parental strain (Figure 8a). Thus, the deletion of nonessential genes did not result in higher energy levels. In fact, the IIG-Bs-27-39 strain maintained the same energy level as the parental strain, despite the redirection of resources toward overflow metabolism. A decrease of ∼25% in the ATP yield was, however, observed during the production phase (Figure 8b), which could be related to the observed overflow metabolism (Figure 7 and Table 2).

**Figure 9:**
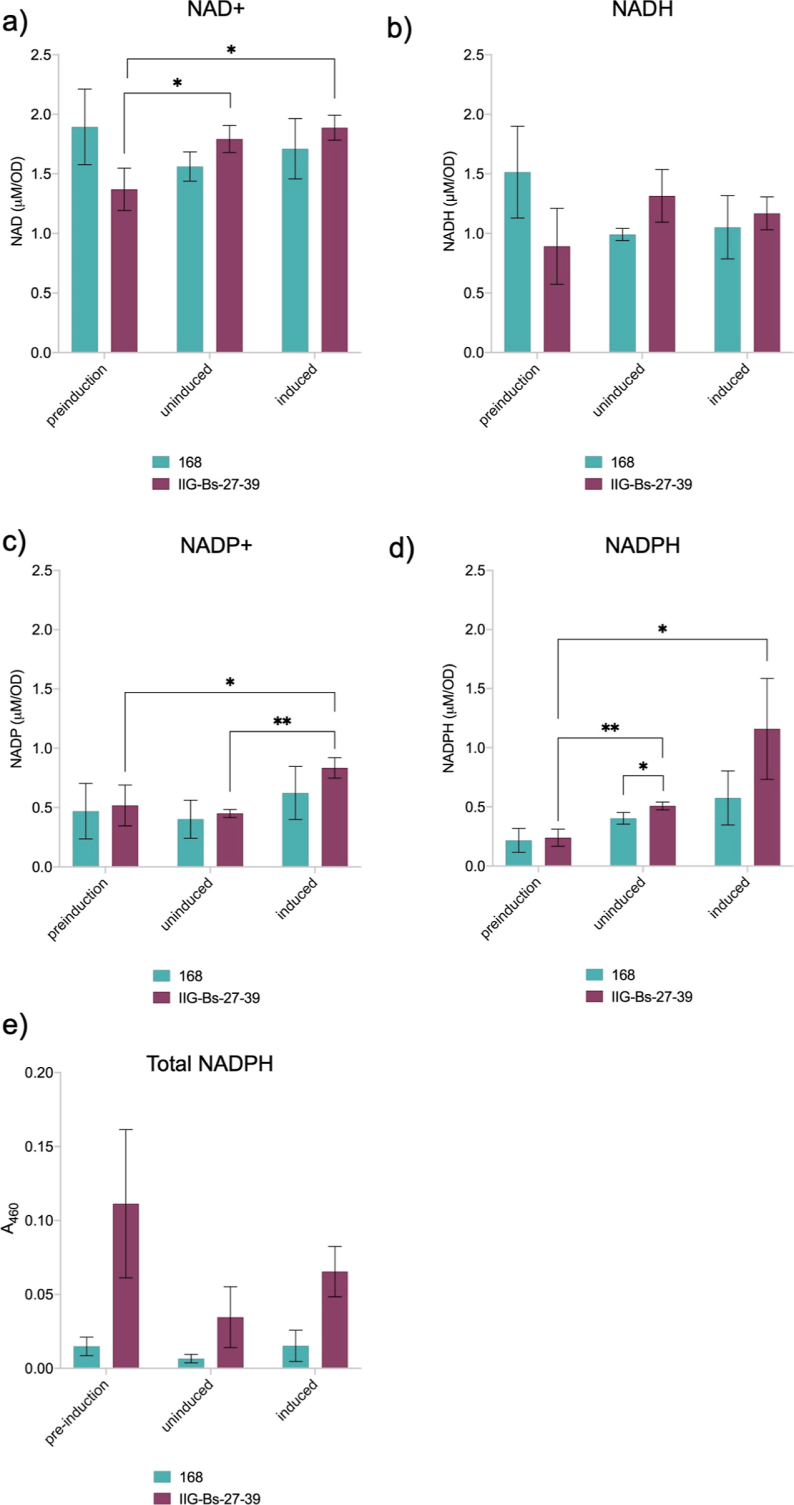
NAD(H) and NADP(H) pools in cells of strains IIG-Bs-27-39 and 168. The *B. subtilis* strains 168 and IIG-Bs-27-39, both carrying the *spaRK* genes and pRAG3::*isaA*, were grown overnight in LB medium. A preculture in M9 medium was started at an OD_600_ value of 0.05. Main cultures were inoculated from the preculture when the preculture reached an OD_600_ value of 1. Samples were withdrawn from the main cultures during the exponential growth phase, prior induction with subtilin at an OD_600_ value of ∼1. Half of the main cultures were subsequently induced, and the other half remained uninduced. Two hours after induction, samples were drawn from both the induced and uninduced main cultures. (a) NAD^+^ and (b) NADH concentrations were measured using the EnzyChrom NAD^+^/NADH Assay Kit (E2ND-100). (c) NADP^+^ and (d) NADPH concentrations were measured using the EnzyChrom NADP^+^/NADPH Assay Kit (ECNP-100). (e) Total cellular NADPH levels were measured using the ABCAM NADPH Assay Kit (ab186031). The data represent the mean values and standard deviation in the measurements for three biological replicates. Student’s *t*-test was used to determine significant changes (*: 0.01 < *p* < 0.05, **: 0.001 < *p* < 0.01).

The amount of NAD(H) and NADP(H) present in a bacterial cell may influence redox balancing and anabolic processes, including protein synthesis. Since the energy charges of the parental strain 168 and the genome-reduced strain IIG-Bs-27-39 were comparable, we wanted to investigate whether a difference in the NAD(H) and NADP(H) pools can explain the superior IsaA yield obtained in the genome-reduced strain IIG-Bs-27-39. To this end, we measured the intracellular NAD^+^, NADH, NADP^+^, and NADPH levels, as well as the total NADPH levels of both strains (Figure 9a–d). Interestingly, per condition, the NAD^+^, NADH, NADP^+^, and NADPH levels of both strains did not differ significantly, except for the NADPH levels of the uninduced cells (Figure 9d). Remarkably, however, statistically significant differences were observed for the NAD^+^, NADP^+^, and NADPH pools of the genome-reduced strain IIG-Bs-27-39 during induction. Since both uninduced and induced cells displayed significant increases in the NAD^+^, NADP^+^, and NADPH concentrations compared to the preinduction condition, these increases might be growth stage-related (Figure 9a,c,d). Nevertheless, these increases were not observed in the parental strain 168. The total cellular NADPH levels also indicated significant differences between the parental strain 168 and the genome-reduced strain IIG-Bs-27-39, where strain IIG-Bs-27-39 tended to display higher total NADPH levels compared to the parental strain 168 under all tested conditions (Figure 9e). Therefore, we conclude that the genome-reduced strain IIG-Bs-27-39 displays a differential regulation of the NAD(H) and NADP(H) pools where, during the production phase, the availability of NAD(H) and NADP(H) is higher than prior induced production. This could, at least in part, be relevant for the enhanced production of IsaA in the IIG-Bs-27-39 strain compared to the 168 strain.

### Protein Functionality

**Figure 10:**
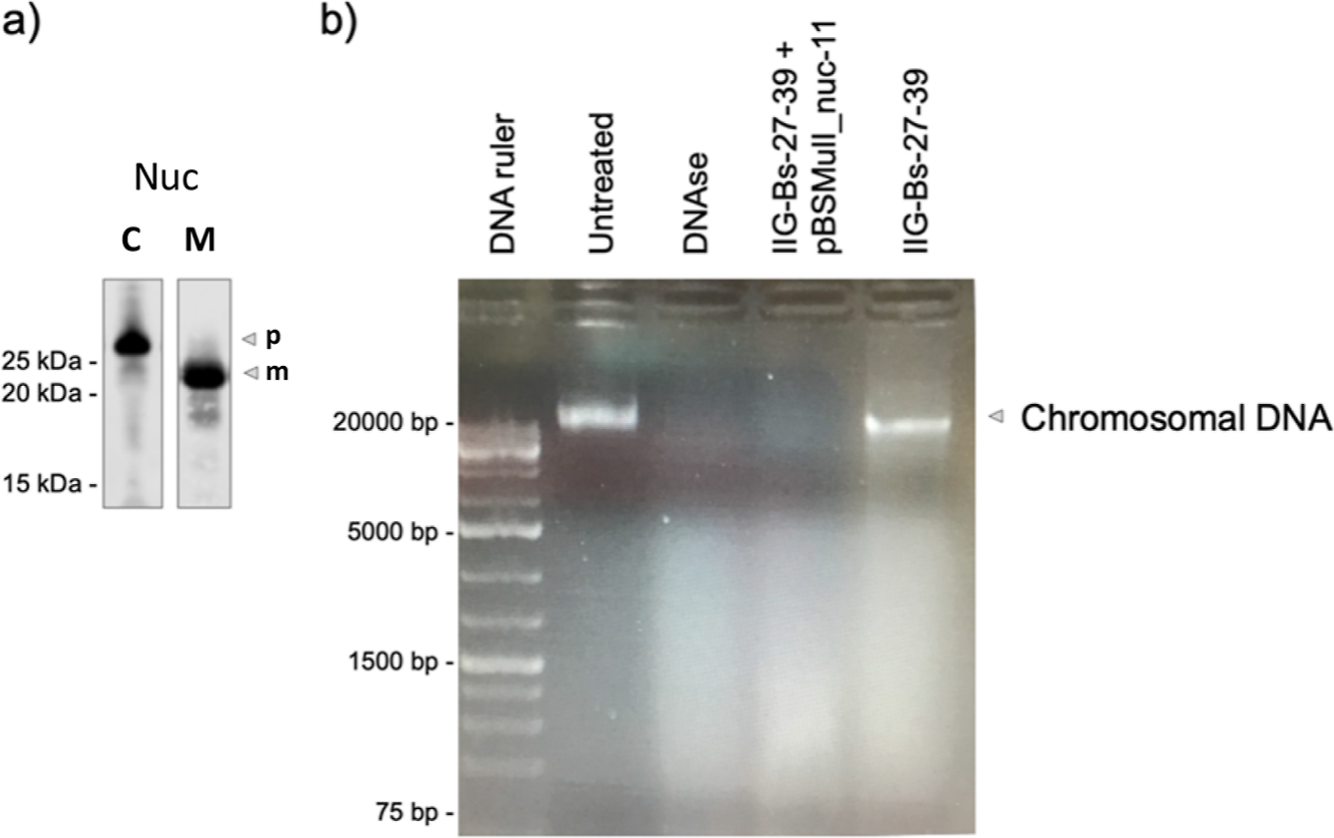
Detection of nuclease activity of the thermonuclease Nuc secreted by the genome-reduced IIG-Bs-27-39 strain. (a) Cells of strain IIG-Bs-27-39 carrying pBSMul-nuc-11 were grown overnight and diluted 1:50 in LB medium. After 8 h of incubation, samples were collected for western blotting and activity analysis. Nuc present in the cell fraction (C) or secreted into the growth medium (M) was detected by western blotting with specific murine antibodies (p, precursor; m, mature protein). (b) To determine the enzymatic activity of Nuc secreted by strain IIG-Bs-27-39, chromosomal DNA from *L. lactis* PA1001 was mixed with spent culture media from strain IIG-Bs-27-39 with or without the pBSMul-nuc-11 plasmid. As a positive control for nuclease activity, the chromosomal DNA was mixed with DNase I. In the negative control, the DNA remained untreated. All samples were incubated for 1 h at room temperature and, subsequently, loaded on a 1% agarose gel to visualize the presence or absence of chromosomal DNA.

To assess whether the IIG-Bs-27-39 strain was able to secrete other heterologous difficult-to-produce proteins in an active state, we selected the staphylococcal thermonuclease Nuc.^6^ The plasmid pBSMulI-nuc-11 encoding Nuc was used to transform the IIG-Bs-27-39 strain. After 8 h cultivation, cell-free culture supernatants were collected to determine the nuclease activity of Nuc on chromosomal DNA from *L. lactis*. The results show that the Nuc protein secreted by strain IIG-Bs-27-39 was processed and actively degraded the chromosomal DNA (Figure 10).

## Discussion

Here, we present the engineered strain IIG-Bs-27-39, lacking 21.6% of the *B. subtilis* 168 genome, as a potential cell factory for heterologous secretory protein production. While previous studies have showcased particular advantages of the use of genome-reduced strains in the production of staphylococcal and other proteins,^6,31,39,40^ this study was focused on exploring the behavior of the IIG-Bs-27-39 strain during fermentation in bioreactors. To pinpoint potential bottlenecks in the secretion of a difficult-to-produce protein, the IIG-Bs-27-39 strain was equipped with the genes for subtilin-inducible expression of the IsaA protein from *S. aureus*. In addition, we verified the capability of strain IIG-Bs-27-39 to produce such proteins with the thermonuclease Nuc from *S. aureus*. The results show that the IIG-Bs-27-39 strain has improved features in terms of growth kinetics and protein production capacity compared to its parental strain 168.

For several reasons, strain IIG-Bs-27-39 was selected from a set of genome-reduced strains of *B. subtilis*. In the first place, because it lacks the genes for eight major extracellular proteases, the low level of extracellular protease production was underpinned by the adequate stability of the staphylococcal proteins CHIPS, IsaA, Nuc, and SCIN in the spent growth medium of the IIG-Bs-27-39 strain. This is consistent with our previous observation that deletion of the *wprA* gene and other protease genes is elementary for production and secretion of IsaA in the genome-minimized mini*Bacillus* strain.^6^ Second, the IIG-Bs-27-39 strain lacks several sporulation and spore germination genes, which makes it attractive not only in terms of biological containment but also because it will not direct cellular resources to these processes. Last but not least, strain IIG-Bs-27-39 was selected for the present study because of its ability to grow well in minimal media, which was essential to assess various physiological parameters. Intriguingly, the choice of minimal medium was relevant for the efficiency of IsaA secretion, as shown by induced IsaA production in the IIG-Bs-27-39 strain grown in M9, PMM, or SMM. While IsaA accumulated in cells grown on PMM or SMM, it was effectively secreted by cells grown in M9. How the medium composition impacts on IsaA secretion is presently not clear, but the main difference is the use of citrate in PMM and SMM, whereas citrate is absent from M9. Citrate can be used directly in the tricarboxylic acid cycle, so, in principle, the less efficient secretion of IsaA should not relate to a lack of energy. Furthermore, citrate chelates iron, thereby impacting on iron acquisition and global redox regulation. Thus, it seems most likely that citrate impacts on the expression of one or more secretion machinery components or that citrate complexes metal ions needed in the post-translocational folding of proteins.^41^ These ideas should be addressed in future studies.

When the performance of strain IIG-Bs-27-39 and the parental strain was compared in the bioreactor, the IIG-Bs-27-39 strain showed a higher specific growth rate. Importantly, the specific growth rate of the parental strain as measured in our study was in line with other reports.^42^ The improved growth characteristics make strain IIG-Bs-27-39 preferable over other genome-minimized *Bacillus* strains, like strain BSK814G2,^43,44^ and the parental strain. One reason why strain IIG-Bs-27-39 performs better than strain 168 with respect to growth and biomass yield could be the removal of prophages and other lytic elements as was previously proposed for *P. putida*.^17,18,23^ Likewise, the deletion of multiple lytic elements was previously shown to increase the biomass yield of *B. subtilis* 168.^45^ Nonetheless, some lysis of strain IIG-Bs-27-39 was still detectable, judging by the extracellular detection of the cytoplasmic marker protein TrxA, but it was clearly reduced compared to strain 168 grown. Of note, residual lysis may explain why some degradation of IsaA was observed in the growth medium of strain IIG-Bs-27-39, as lysis would release cytoplasmic proteases.^6,39^ In any case, the increased growth rate and biomass yields of strain IIG-Bs-27-39 appear to be an interesting characteristic for large-scale fed-batch fermentation at high cell densities.

Analysis of the amino acid pools in the IIG-Bs-27-39 strain did not reveal particular limitations compared to the parental strain, and the amino acid pools of strain IIG-Bs-27-39 even increased in the production phase. At present, we cannot say whether this effect is due to enhanced amino acid synthesis or intracellular protein turnover as has been previously reported.^46^ Even though alanine was the second most abundant amino acid in both strains in our present study, the measured values were lower compared to other previous reports. This could be related to differences in the glucose concentration used in our and other studies, or the consumption of alanine during the filtration step.^43,47−50^ Nonetheless, it seems unlikely that alanine was present in limiting amounts because alanine was still present in excess over all other amino acids except glutamate. An interesting possibility that remains to be explored in the IIG-Bs-27-39 strain is the deletion of the *rocDEF*-*rocR* genes, which would set a limit to arginine catabolism. This was previously shown to enhance cell yields and boost protein production through an altered glutamate metabolism.^51^ Alternatively, the deletion of *rocG* in combination with modification in the cultivation conditions has been shown to improve protein production in genome-reduced *Bacillus*.^52^ This effect is probably related to an increase in the conversion of 2-oxoglutarate to glutamate.^52^

The higher levels of overflow metabolites, such as acetate and acetoin, secreted by strain IIG-Bs-27-39 represent a spill of carbon that is not incorporated in the desired protein product. One possibility to prevent the deviation of carbon toward overflow metabolism could be to reduce the growth rate. On the other hand, the parental strain excreted higher levels of pyruvate than the IIG-Bs-27-39 strain, which also represents a loss of carbon. However, the excreted pyruvate could later be taken up again and used in the Krebs cycle for the generation of NAD(P)H. A ^13^C metabolic flux analysis, possibly combined with a transcriptome analysis, could be helpful to elucidate the reasons why strain IIG-Bs-27-39 excretes overflow metabolites.

The elimination of nonessential genes in strain IIG-Bs-27-39 had no major effects on the cellular energy levels, as indicated by the ATP yields and the AEC, which were comparable to those measured for the parental strain. However, we did observe a ∼25% decrease in the ATP yield during the production phase in the IIG-Bs-27-39 strain. This could be related to a previous observation made with the related strain IIG-Bs-27-47-24, which showed a strong increase in the levels of proteins related to translation and protein synthesis when IsaA expression was induced.^36^ Most likely, this is also the case in strain IIG-Bs-27-39, since judged by the comparable properties of strains IIG-Bs-27-47-24 and IIG-Bs-27-39 in IsaA production, the translational capacity of both strains is probably very similar. If so, it seems plausible that strain IIG-Bs-27-39 consumes more ATP in the production phase for synthesis of proteins involved in translation and protein synthesis, which are energetically very expensive processes.^53,54^ In addition, the enhanced synthesis of proteins could lead to additional ATP consumption by major chaperones, like DnaK and GroEL/ES, which guide the correct folding of newly synthesized proteins and require large quantities of ATP.^55^ Of note, although the ATP yields and energy charge of strains IIG-Bs-27-39 and 168 are very similar, this does not necessarily mean that both strains produce and consume ATP at the same rates. For instance, the loss of resources by overflow metabolism in strain IIG-Bs-27-39 could imply that this strain produces more ATP than the parental strain but also consumes more ATP. In this respect, our observation that the genome-reduced strain IIG-Bs-27-39 displayed increases in the NAD^+^ and NADP(H) pools in the production phase and elevated levels of the total cellular NADPH in all tested conditions seems relevant, because it implies that the IIG-Bs-27-39 strain has more energy available for anabolic processes. Combined with the other beneficial features for secretory protein production, the elevated levels of NADPH will contribute to the superior production of IsaA by the IIG-Bs-27-39 strain.

Last, our study shows that strain IIG-Bs-27-39 is capable of producing and secreting fully functional heterologous proteins, as exemplified with the thermonuclease Nuc from *S. aureus*. This underscores our view that the IIG-Bs-27-39 strain forms an attractive chassis for further development of metabolically efficient and highly productive cell factories that can deliver high-quality secreted proteins for industrial and biomedical applications.

## Conclusions

In the present study, we have benchmarked the genome-reduced *B. subtilis* strain IIG-Bs-27-39 against its parental strain 168 with respect to heterologous protein production and metabolic parameters during bioreactor cultivation. The IIG-Bs-27-39 strain presented superior production of the staphylococcal antigen IsaA, and higher specific growth rates and biomass yields. At the metabolic level, changes in byproduct formation and internal amino acid pools were observed, whereas energetic parameters such as the ATP yield, ATP/ADP levels, and AEC were comparable between the two strains. Intriguingly, however, we observed significant differences in the NAD^+^ and NADP(H) pools, which indicate that the IIG-Bs-27-39 strain has more energy available for anabolic processes and protein production, thereby providing a link between strain physiology and production performance. Altogether, we conclude that the genome-reduced strain IIG-Bs-27-39 represents an attractive chassis for future biotechnological applications.

## Materials and Methods

### Strains and Plasmids

Strains and plasmids used in this study are listed in Table 1. *B. subtilis* 168 *spaRK* was made *trp*^+^ as previously described.^56^*E. coli* JM109 was employed as a cloning host to obtain derivatives of plasmid pJOE6743.1 needed to generate the IIG-Bs-27-39 strain. *B. subtilis* strain ATCC 6633 was used to produce subtilin, which was required to induce the expression of IsaA.

### Molecular Biology Techniques

Molecular cloning was carried out according to standard protocols. *E. coli* JM109 was transformed by a one-step method based on the use of a single transformation and storage solution.^66^*B. subtilis* strains were genetically transformed according to the Spizizen protocol during the initial stages of genome reduction.^67^ Since the transformation efficiency was diminished upon the successive deletion of several gene clusters, a mannitol-inducible *comKS* cassette was introduced during the construction of the *B. subtilis* IIG-Bs27-39 strain.^68^ Competence of strains containing the *comKS* cassette was induced by the addition of 0.5% mannitol as described before.^68^ Plasmid pBSMulI-nuc-11 was constructed by conventional cloning techniques as described in Data S1.

### Construction of IIG-Bs-27-39

The presently applied strain IIG-Bs-27-39 represents an intermediate step in the previous *B. subtilis* 168 genome reduction effort that led *via* the *B. subtilis* strain IIG-Bs20 (lacking 13.5% of the genome^59^ to the mini*Bacillus* strain).^43^ To develop strain IIG-Bs-27-39 from strain IIG-Bs-20, a markerless gene deletion system was used that is based on the mannose phosphoenolpyruvate-dependent phosphotransferase system.^59^ In short, the upstream and downstream regions (approximately 0.7 Kb), flanking the target region that was to be deleted, were joined and inserted into the pJOE6743.1 plasmid, which carries a spectinomycin resistance marker and the P*manP*-*manP* cassette. Spectinomycin-resistant *B. subtilis* transformants were selected on LB agar plates with 100 μg/mL spectinomycin and, subsequently, a counter-selection was performed by growing the transformants in LB medium with 0.5% mannose. Last, colonies were selected on LB agar plates with 0.5% mannose.^59^ The IIG-Bs-27-39 strain thus obtained has a genome of 3.11 Mb, lacking 26.1% of the genome of strain 168. A detailed overview of the deleted regions in strain IIG-Bs-27-39 is provided in Table S1. The *spaR* and *spaK* genes were introduced in the *amyE* locus of strain IIG-Bs-27-39 and other genome-reduced strains with the integrative plasmid pNZ8900, which was necessary for subtilin-inducible expression of IsaA from plasmid pRAG3::*isaA*.

### Genome Sequencing

Next-generation sequencing was used to determine the genome sequence of strain IIG-Bs-27-39 carrying the *spaRK* genes in the *amyE* locus and plasmid pRAG3::*isaA*. DNA extraction, quantification, and sequence analysis were performed as described before, but *de novo* assembly of paired-end reads was performed using CLC Genomics Workbench v20 (QIAGEN, Hilden, Germany).^6^ The sequence of the IIG-Bs-27-39 strain is available at http://www.subtiwiki.uni-goettingen.de/v4/minibacillus?page=2&page_size=50.^69^

### Western Blotting

Bacterial culture samples (∼1.5 mL) were centrifuged to separate the cells and the growth medium. The proteins in the growth medium were precipitated with 10% (w/v) trichloroacetic acid (TCA). After TCA precipitation, proteins were resuspended in lithium dodecyl sulfate (LDS) gel loading buffer (Life Technologies). Proteins from the cell pellet fraction were extracted by bead-beating using a Precellys24 tissue homogenizer. The samples were incubated at 95 °C for 10 min. Proteins were loaded on 10% precast Bis-Tris NuPage gels (Invitrogen). Before loading, the samples were corrected to an OD_600_ value of 2.0. After LDS-PAGE, proteins were transferred onto a nitrocellulose membrane by semidry blotting (Amersham Protran 0.45 μm, GE Health Care Sciences). 5% (w/v) skim milk was used to block the membranes. Phosphate-buffered saline plus Tween 20 (PBS-T) was used to wash the membranes several times. Next, the membranes were incubated with a polyclonal rabbit antibody (1:5000) or a human monoclonal antibody for 1 h at room temperature. The membranes were then washed with PBS-T and incubated with a secondary goat-anti rabbit/human IgG-IRDye800 CW conjugate (1:5000; LI-COR). Last, the membranes were washed with PBS-T and subsequently PBS. Protein bands with the bound secondary antibody were visualized using an Amersham Typhoon Biomolecular imager. Prestained Precision Plus protein markers were purchased from Bio-Rad (Hercules, USA).

### Protein Stability Test

To assess the stability of staphylococcal proteins in spent growth media of different genome-reduced *B. subtilis* strains, we used the chemotaxis inhibitory protein CHIPS, the immunodominant protein IsaA, the thermonuclease Nuc, and the staphylococcal complement inhibitor SCIN as previously described.^6^ Briefly, the four staphylococcal proteins were expressed in *L. lactis* from the nisin-inducible plasmids pNG4210::*chp*, pNG4210::*isaA*, pNG400::*nuc*, or pNG4210::*scn*. After 16 h of induced production, cell-free culture supernatants containing either CHIPS, IsaA, Nuc, or SCIN were obtained by centrifugation. Aliquots of these supernatants were mixed with spent cell-free culture supernatants of different genome-reduced *B. subtilis* strains, which had been grown overnight. After incubation for 2 h at 37 °C, proteins were precipitated with 10% trichloroacetic acid (TCA) to assess the stability of each staphylococcal protein by LDS-PAGE and western blotting with specific antibodies against IsaA, SCIN, Nuc, or the C-terminal his-6 tag on CHIPS.

### Media Composition

For transformation and initial precultures, *Bacillus* strains were grown in Lysogeny broth (LB) containing 10 g/L tryptone, 5 g/L yeast extract, and 10 g/L NaCl. For stable plasmid maintenance, the LB medium was supplemented with erythromycin 2 μg/mL, kanamycin 20 μg/mL, or spectinomycin 100 μg/mL if appropriate. LB plates contained 15 g/L agar. All cultivations were conducted at 37 °C with vigorous shaking.

The M9 minimal medium used for secondary precultures and main cultures in bioreactors contained per liter of nanopure water: 1 g of NH_4_Cl, 0.5 g of NaCl, 8.5 g of Na_2_HPO_4_·H_2_O, and 3.0 g of KH_2_PO_4_. The pH was adjusted to 7.0 using 4 M NaOH. The following components were sterilized separately and then added (per liter of final medium):^47^ 246 mg of MgSO_4_, 14.7 mg of CaCl_2_·2H_2_O, 0.013.5 mg of FeCl_3_·6H_2_O, 30 mg of 3,4-dihydroxybenzoic acid, 1 mg of MnCl_2_·4H_2_O, 1.7 mg of ZnCl_2_, 0.43 mg of CuCl_2_·2H_2_O, 0.60 mg of CoCl_2_·6H_2_O, and 0.60 mg of Na_2_MoO_4_·2H_2_O. Glucose was used as the only carbon source at a final concentration of 5 g/L for the precultures. When growing cells in M9 minimal medium, no antibiotics were added. The MBU, Spizizen’s (SMM), and *Bacillus pumilus* (PMM) minimal media were prepared as described before.^67,68,70,71^ When required, subtilin was prepared in the same minimal medium as the cultures to be induced.^35^

### Culturing in Shake Flasks and Bioreactors

*B. subtilis* cells from a cryo-stock were grown overnight on LB agar plates supplemented with kanamycin and erythromycin. For the first preculture, single colonies were picked and used to inoculate 10 mL of LB medium in 100 mL baffled shake flasks. Cultures were incubated on a rotary shaker at 37 °C and 230 rpm (Multifors, Infors AG, Switzerland). Exponentially growing cultures with an optical density at OD_600_ values between 0.5 and 1.5 were diluted 1:5000 in 50 mL of M9 minimal medium in 500 mL baffled shake flasks to start a second preculture. These cultures were grown to exponential phase, and cells were harvested by centrifugation (5 min, 10,000*g*). The pelleted cells were washed with fresh M9 medium without glucose, resuspended in the same medium, and used to inoculate 300 mL M9 medium without glucose in 1 L bioreactors to an OD_600_ value of 0.1. To this end, a DASGIP Bioblock setup with 4 independently running bioreactors was applied (DASGIP, Jülich, Germany). The bioreactors were operated at 37 °C, pH 7.1, an aeration rate of 9 L/h, and a stirring speed of 1000 rpm. Foam formation during production was suppressed with filter-sterilized antifoam. When the cultures reached an OD_600_ value of 1.0, the production of IsaA was induced with 1% subtilin, which was prepared in advance as described previously, but by growing *B. subtilis* ATCC6633 in M9 minimal medium.^42^ Aliquots were taken from the bioreactors at different time points for further analysis. All bioreactor experiments involved two biological replicates. The latter was decided considering the high reproducibility of duplicate bioreactor fermentations previously demonstrated by us^17,56,72−75^ and others^76−79^ using the same experimental setup. In addition, four operating reactors appear to be the maximum to ensure fast sampling and high-quality sample processing (including quenching, extraction, and storage), given the various analyses run in parallel. In contrast to analyses of proteome and transcriptome with time corridors for processing in the range of seconds to minutes, metabolome analyses, as performed in the present studies, are particularly dependent on trouble-free, rapid handling due to metabolite time constants down to the millisecond range.^79−81^ As shown, all data throughout this work exhibited high precision and accuracy to enable quantitative comparison of the two strains.

### Dry Cell Weight

Cell concentration was measured as OD_600_. To determine the dry cell weigh (DCW), aliquots of culture broth from the bioreactors were filtered using vacuum filtration on dried and preweighted membrane filters (0.2 μm pore size, RC membrane filters, Sartorius, Göttingen, Germany). Then, cells were washed on the filter with 10 mL of isotonic NaCl solution and consecutively with deionized water. The filters were dried at 80 °C until constant weight. OD_600_ and DCW correlated with a coefficient factor of 1 OD_600_ = 0.4155 g_DCW_/L (Figure S1).

### Intracellular Amino Acids

To measure intracellular amino acids, samples were collected and processed as described previously.^48^ In short, 2–10 mL of cell suspension was harvested *via* vacuum filtration in cellulose-nitrate filters (0.2 μm pore size, 47 mm, Sartorius, Göttingen, Germany). The cells were washed immediately on the filter with 1.15% NaCl solution mimicking the ionic strength of the growth medium. Filters carrying the cells were incubated for 15 min at 100 °C in water with the internal standard α-aminobutyrate. Subsequently, the samples were cooled down on ice and recovered from the filter for quantification by high-performance liquid chromatography (HPLC; Agilent 1200, Waldbronn, Germany) using a reversed-phase column (Gemini 5 μm, 150 × 4.6 μm, Phenomenex, Aschaffenburg, Germany).^82^

### Quantification of Glucose and Organic Acids

To quantify glucose and organic acids, 2 mL of culture was taken from the bioreactors, and cell-free supernatants were obtained by centrifugation. The glucose concentration was quantified with a glucose analyzer (YSI Life Sciences, Yellow Springs, USA). Meanwhile, pyruvate, acetate, succinate, lactate, formate, acetoin, 2,3-butanediol, isobutyrate, ethanol, and isovalerate were measured by a HPLC equipped with UV (210 nm) and refractive index detectors (Hitachi, Tokyo, Japan). To this end, isocratic elution with 12 mM H_2_SO_4_ was performed using an Aminex HPX-87H column (Bio-Rad, Hercules, USA) at 45 °C and a flow rate of 0.5 mL/min.

### Adenylate Energy Charge

For determination of the AEC, aliquots of 5–25 mL of cell culture were cooled down immediately with liquid nitrogen to arrest metabolism^47^ before applying the fast filtration method.^83^ To this end, cells collected on a filter (pore size 0.45 μm, 47 mm, S-Pak filters, Millipore, Schwalbach, Germany) were washed twice with 0.85% NaCl, and the filter with the cells was placed immediately in ice-cold 60% ethanol for metabolic extraction. Tubes with the samples were submerged in liquid nitrogen for metabolic quenching. Samples were stored at −80 °C until further processing. For processing of the stored samples, they were thawed on ice, vortexed, shaken, and centrifuged (5 min at 4 °C and 15,500*g*), and the supernatant fraction was collected. The extraction step and subsequent centrifugation were repeated, but with cold double-distilled water. Supernatants from the two extraction steps were combined and restocked with double-distilled water to a final organic solution concentration of 10% and stored at −80 °C prior to lyophilization. The lyophilized samples were resuspended in 500 μL of resuspension buffer of the ATP Colorimetric/Fluorometric Assay Kit (Sigma-Aldrich), deproteinized using Vivaspin500 columns (10 kDa), and eluted by centrifugation (30 min at 4 °C and 15,500*g*). The deproteinized samples thus obtained were used for the colorimetric determination of ATP, ADP, and AMP using an ATP Colorimetric/Fluorometric Assay Kit (MAK190, Sigma-Aldrich). The protocol was followed according to the manufacturer’s instructions.

### NAD(H) and NADP(H) Pools

*B. subtilis* strains 168 and IIG-Bs-27-39, both carrying the *spaRK* genes and pRAG3::*isaA*, were grown overnight in LB medium. A preculture in M9 medium with 0.2 g/L tryptophan was started at an OD_600_ value of 0.05. Main cultures in M9 medium with 0.2 g/L tryptophan were started from the preculture when the preculture reached an OD_600_ value of 1. First, samples were collected from the main cultures during the exponential growth phase at an OD_600_ value of ∼1, before the induction of IsaA production with subtilin. Next, in half of the cultures, IsaA production was induced, and the other half of the cultures was kept uninduced. 2 h after induction, samples were withdrawn from both the induced and uninduced main cultures. NAD^+^ and NADH concentrations were measured using the EnzyChrom NAD^+^/NADH Assay Kit (E2ND-100; BioAssay System, Hayward, CA). NADP^+^ and NADPH concentrations were measured using the EnzyChrom NADP^+^/NADPH Assay Kit (ECNP-100; BioAssay System, Hayward, CA). The total cellular NADPH content in the collected samples was measured using the ABCAM NADPH Assay Kit (ab186031). Briefly, the cultures were standardized to an OD_600_ value of 1, and the cells were collected by centrifugation (3 min at 15,500*g*). The supernatants were discarded, and the cell pellets were resuspended in the lysis buffer provided by the kit. 5 mg/mL lysozyme was added to each sample, and the tubes were incubated at 55 °C for 10 min. Tubes were centrifuged to pellet cell debris (2 min at 15,500*g*), and the supernatant was used for the determination of the NADPH content. To this end, 45 μL of this supernatant was added to a clear-bottom 96-well plate. 45 μL of NADPH reaction mixture was added to the samples, and the samples were incubated for 1 h in the dark. After 1 h, the absorbance was measured at 460 nm.

### Determination of Nuc Activity

To assess the functionality of heterologous proteins produced by the IIG-Bs-27-39 strain, the staphylococcal thermonuclease Nuc was applied as a model protein. To this end, the plasmid pBSMulI-nuc-11 was used to transform the IIG-Bs-27-39 strain, which results in constitutive Nuc expression. Upon overnight cultivation in LB, the IIG-Bs-27-39 cells carrying pBSMulI-nuc-11 were diluted 1:50 in fresh LB and further incubated for 8 h. At this point, cells were harvested by centrifugation to obtain cell-free culture supernatants containing Nuc. Strain IIG-Bs-27-39 without the pBSMulI-nuc-11 plasmid was treated in the same way as a control. To determine the nuclease activity, 10 μL of chromosomal DNA from *L. lactis* PA1001 were mixed either with 10 μL of spent culture media or with 200 ng of recombinant DNase I (QIAGEN) as a positive control for nuclease activity. Untreated chromosomal DNA was used as a negative control. Samples were incubated for 1 h at room temperature, and enzyme activity was stopped by the addition of DNA-loading buffer. Samples were loaded on a 1% agarose gel to visualize degradation of the chromosomal DNA by Nuc.^65^

### Statistical Analyses

General statistical analyses were performed with GraphPad Prism version 9, and *p*-values <0.05 were considered to indicate statistical significance.

## Supporting Information Available

Construction of the pBSMul1-nuc-11 plasmid; correlation between optical density at 600 nm (OD_600_) and DCW (g/L) for *B. subtilis* 168 and IIG-Bs-27-39; CO_2_ evolution [mM/g/h] in bioreactor cultures of strain IIG-Bs-27-39 and the parental strain 168; gene deletions in genome-reduced *B. subtilis* strains (PDF)
