# Supplementary material for: Metabolic Profile of the Genome-Reduced Bacillus subtilis Strain IIG-Bs-27-39: An Attractive Chassis for Recombinant Protein Production
Source: ACS Synth Biol. 2024 Jul 9;13(7):2199–214. doi: 10.1021/acssynbio.4c00254 (PMC11264325; doi:10.1021/acssynbio.4c00254)
Supplement: Supplementary file 1 — sb4c00254_si_001.pdf [file sb4c00254_si_001.pdf]

Supporting Information

Metabolic profile of the genome-reduced *Bacillus subtilis* strain IIG-Bs-27-39, an attractive chassis for recombinant protein production

Rocío Aguilar Suárez[1], Michael Kohlstedt[#2], Ayşegül Öktem[#1], Jolanda Neef[1], Yuzheng Wu[3], Kaiya Ikeda[3], Ken-ichi Yoshida[3], Josef Altenbuchner[4], Christoph Wittmann[2], Jan Maarten van Dijl[1*]

#These authors contributed equally

[1] University Medical Center Groningen-University of Groningen, Department of Medical Microbiology, 9700RB Groningen, The Netherlands

[2] Institute for Systems Biotechnology, Saarland University, 66123 Saarbrücken, Germany

[3] Department of Science, Technology and Innovation, Kobe University, Kobe 657-8501, Japan

[4] Institute for Industrial Genetics, University of Stuttgart, 70569 Stuttgart, Germany

*Correspondence: Jan Maarten van Dijl, University Medical Center Groningen, Department of Medical Microbiology, Hanzeplein 1, 9700RB Groningen, the Netherlands, tel. +31-50-3615187, e-mail: j.m.van.dijl01@umcg.nl

Supplementary Data S1. Construction of the pBSMul1-nuc-11 plasmid.

The plasmid pBSMul1-nuc-11 was created for constitutive production of Nuc and to assess the activity of the secreted Nuc by the *B. subtilis* strain IIG-Bs27-39-1. The plasmid pBSMul1-cut-11 was used as backbone for the insertion of *nuc*. To this end, the oligonucleotides pBSMul1_NotI_F (5' TAATTAGCGGCCGCACTTATCGGCTCTGATGGCG 3') and pBSMul1_EcoRV_R (5' GCCGCCAGGATATCTCCTCCTTATTATGTAAATCGCTCC 3') were used to amplify the backbone without the cutinase fragment and to introduce the *Not*I and *Eco*RV cleavage sites. The sequence of SP_{xyna}-*nuc* was amplified from the plasmid pRAG3::*nuc* with the oligonucleotides nuc_EcoRV_F (5' ATTGAAAGGAGGTGGATATCATGTTTAAGTTTAAA 3') and nuc_NotI_R (5' TAATGTGCGGCCGCTTATTGAC 3'), which also added the *Not*I and *Eco*RV restriction enzyme sites. The oligonucleotides were obtained from Eurogentec. The amplified PCR products were digested with EcoRV and NotI, and T4-ligated. Phusion-HF DNA polymerase, restriction endonucleases, and T4 DNA ligase were obtained from New England Biolabs. *E. coli* DH5-α cells were used as cloning intermediate. The resulting plasmid was verified by Sanger sequencing (Eurofins Genomics) and named pBSMul1-nuc-11.

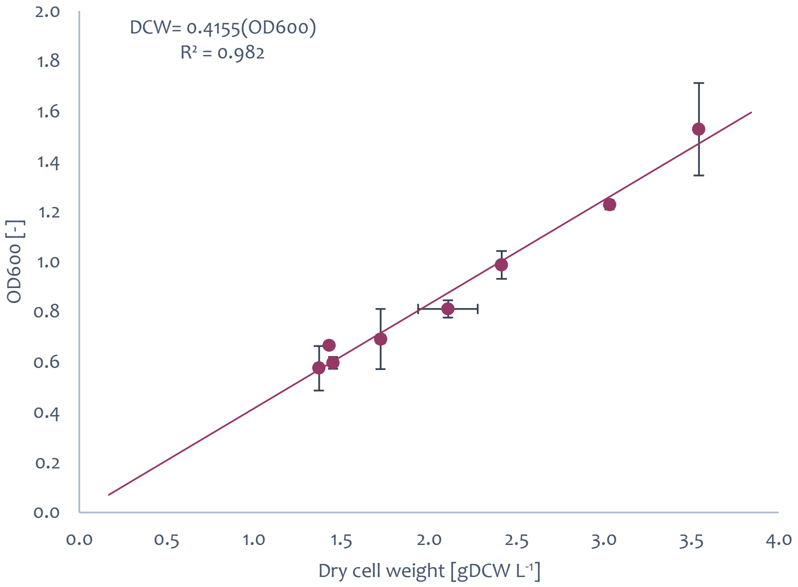

Supplementary Figure S1. Correlation between optical density at 600 nm (OD_{600}) and dry cell weight (g/L) for *B. subtilis* 168 and IIG-Bs-27-39. Strain IIG-Bs-27-39 and the parental strain 168, both carrying the *spaRK* genes and plasmid pRAG3::*isaA*, were grown in batch cultures in M9 minimal medium with 5 g/L glucose as sole carbon source, and the correlation between optical density at 600 nm (OD_{600}) and dry cell weight (g/L) was measured. The correlation is similar for both strains with a coefficient factor of 1 OD_{600}= 0.4155.gDCW/L.

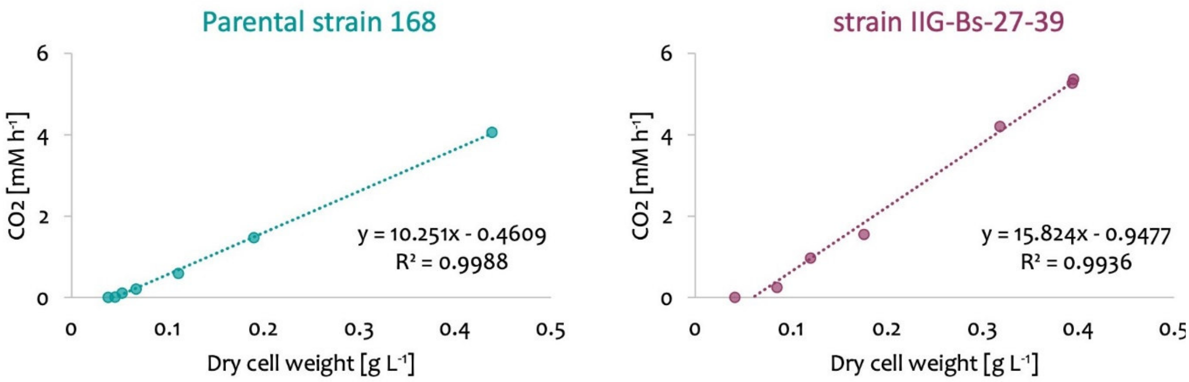

Supplementary Figure S2. CO_2 evolution [mM/g/h] in bioreactor cultures of strain IIG-Bs-27-39 and the parental strain 168. Strain IIG-Bs-27-39 and the parental strain 168, both carrying the *spaRK* genes and plasmid pRAG3::*isaA*, were grown in batch cultures in M9 minimal medium with 5 g/L glucose as sole carbon source.

Supplementary Table S1: Gene deletions in genome-reduced *Bacillus subtilis* strains

Strain name	Characteristic	Deleted Genes
IIG-Bs-1	Trp+	
IIG-Bs-2	Δ*manPA::erm*	*manP, manA*
IIG-Bs-3	ΔproΦ2 (ICEBs1)	*ydcL, immA, immR, sacV, ydzL, ydcO, ydcP, ydcQ, ydcR, ydcS, ydcT, yddA yddB, yddC, yddD, yddE, yddF, yddG, yddH, yddI, yddJ, yddK, rapI, phrI, yddM*
IIG-Bs-4	ΔproΦ5	*ynxB, ynzFG, ynaB, ynaC, ynaD, ynaE, ynaF, ynaG, ynzI, ynaI*
IIG-Bs-5	ΔproΦ6	*yozV, yobD, yozH, yozI, yobE, yobF, yozW, yozX, yozJ, yozY, yozZ, rapK, phrK, yobH, yozK, yozL, yozM, yobI, yoyA, yobJ, yobK, yobL, yobM, yobN, yobO*
IIG-Bs-8	ΔCm^R	Cm^R
IIG-Bs-9	ΔSubtilosin	*sboA, sboX, albA, albB, albC, albD, albE, albF, albG*
IIG-Bs-10	ΔPlipastin	*ppsA, ppsB, ppsC, ppsD, ppsE*
IIG-Bs-11	ΔBacilysin	*bacA, bacB, bacC, bacD, bacE, bacF, ywfH*
IIG-Bs-12	ΔproΦ7	*yrkK, yrkJ, yrkI, yrkH, yrkF, yrkE, yrkD, yrzM, yrzN, yrkC, yrkB, bltR, blt, bltD, yrkA, yrzO, yrdR, yrdQ, yrdP, czcD, yrdN, gltR, yrdK, brnQ, azlB, azlC, azlD, yrdF, cypA, yrdD, yrdC, yrdB, yrdA, aadK, yrpB, yrpC, yrpD, yrpE*
IIG-Bs-13	Bacilysocin	*ytpA, ytpB, ytoA*
IIG-Bs-14	*Bacillus* toxin	*sdpA, sdpB, sdpC, sdpI, sdpR*
IIG-Bs-20	Protease, sporulation (*sigE, sigG*)	*bpr, spoIIGA, sigE, sigG*
IIG-Bs-22	*ydgG-ydhU;* glucomannan utilisation, cell wall synthesis	*ydgG, ydgH, ydgI, ydgJ, ydgK, ydhB, ydhC, ydhD, ydhE, ydhF, phoB, ydhG, ydhH, ydhI, ydhJ, ydhK, pbuE, gmuB, gmuA, gmuC, gmuD, gmuR, gmuE, gmuF, gmuG, ydhU*
IIG-Bs-23	*spoIIAA-spoVAF;* processing of *sigF*	*spoIIAA, spoIIAB, sigF, spoVAA, spoVAB, spoVAC, spoVAD, spoVAEB, spoVAEA, spoVAF*
IIG-Bs-26	*epsO-slrR;* biofilm	*epsO, epsN, epsM, epsL, epsK, epsJ, epsI, epsH, epsG, epsF, epsE, epsD, epsC, epsB, epsA, slrR*
IIG-Bs-27	*yqxM-tasA;* biofilm	*yqxM, sipW, tasA*
IIG-Bs-27-1	*glcP-yhjM;* 3,3´-neotrehalosadiamine, kanosamine	*glcP, ntdC, ntdB, ntdA, yhjM*
IIG-Bs-27-2	*lytD-ywtE;* germination, autolysin	*lytD, yvyI, gerBA, gerBB, gerBC, ywtG, ywtF, ywtE*
IIG-Bs-27-3	*yqeF-yrkS;* sporulation, sigma factor, skin element	*yqeF, cwlH, yqeD, yqeC, yqeB, spoIVCB, spoIVCA, arsC, arsB, yqcK, arsR, yqcI, rapE, phrE, yqzI, yqcG, yqcF, yqxJ, yqxI, cwlA, yqxH, yqxG, yqcE, yqcD, yqcC, yqcB, yqcA, yqbT, yqbS, yqbR, yqbQ, yqbP, yqbO, yqbN, yqdB, yqbM, yqbK, yqzN, yqbJ, yqbI, yqbH, yqbG, yqbF, yqbE, yqbD, yqbC, yqbB, yqbA, yqaT, yqaS, yqaR, yqaQ, yqaP, yqaO, yqzO, yqaM, yqaL, yqaK, yqaJ, yqaI, yqaH, yqaG, yqdA, yqaF, yqaE, yqaD, yqaC, yqaB, spoIIIC, yrkS*
IIG-Bs-27-4	flagella (deletion without promotor and sigD)	*fliE, fliF, fliG, fliH, fliI, fliJ, ylxF, fliK, flgD, flgE, ylzI, fliL, fliM, fliY, cheY, fliZ, fliP, fliQ, fliR, flhB, flhA, flhF, ylxH, cheB, cheA, cheW, cheC, cheD*
IIG-Bs-27-5	*spoIVB;* sporulation	*spoIVB*
IIG-Bs-27-7	*gerPF-wprA;* germination, protease	*gerPF, gerPE, gerPD, gerPC, gerPB, gerPA, yisI, yisJ, yisK, yisL, wprA*
IIG-Bs-27-8	*yvzG-fliK;* flagella	*yvzG, fliT, fliS, fliD, yvyC, hag, csrA, fliW, yviE, flgL, fliK*

IIG-Bs-27-9	*cotO-spoVIF;* spore proteins	*cotO, cotZ, cotY, cotX, cotW, cotV, yjcA, yjzK, yjcZ, spoVIF*
IIG-Bs-27-11	*yclG-yczG;* germination, regulatory genes for differentiation	*yclG, gerKA, gerKC, gerKB, yclH, yclI, yclJ, yclK, rapC, phrC, yczM, yczN, yclM, yclN, yclO, yclP, yclQ, ycnB, ycnC, ycnD, ycnE, yczG*
IIG-Bs-27-14	*yybP-yyaJ*	*yybP, yybO, yyzI, yyzJ, yyzK, yyzL, yybN, yybM, yybL, yybK, yybJ, yybI, yybH, yybG, yybF, yybE, yybD, yybC, yybB, yybA, yyaT, yyaS, yyaR, yyaQ, yyaP, tetB, tetL, yyaO, yyaN, yyaM, yyaL, yyaK, yyaJ*
IIG-Bs-27-16	*yitM-yitS;* protease	*yitM, yitO, yitP, yizB, yitQ, yitR, nprB, yitS*
IIG-Bs-27-17	protease	*vpr*
IIG-Bs-27-18	*yxxB-yxcD;* inositol degradation	*yxxB, yxeR, yxeQ, yxeP, yxeO, yxeN, yxeM, yxeL, yxeK, yxeJ, yxeI, yxeH, yxeG, yxeF, yxeE, yxeD, yxeC, yxeB, yxeA, yxdM, yxdL, yxdK, yxdJ, iolJ, iolI, iolH, iolG, iolF, iolE, iolD, iolC, iolB, mmsA, iolR, iolS, yxcE, yxcD*
IIG-Bs-27-19	*yktA-nprE;* protease	*yktA, yktB, ykzI, suhB, ykzC, ykzD, nprE*
IIG-Bs-27-20	*ywbB-epr;* protease	*ywbB, ywbA, epr*
IIG-Bs-27-24	integration of P_{mtlA}-*comK-comS* between *hisI-yvcB*	Partial deletion of *yvcA*
IIG-Bs-27-25	*yydD/phrG;* regulatory genes for differentiation	*yydD, yydC, yydB, yydA, yyzF, yycS, yycR, yycG, yycQ, yycP, yycO, yycN, rapG, phrG*
IIG-Bs-27-26	*mrp-ybfJ*; protease	*mpr, ybfJ*
IIGBs-27-27	*kinD-motB;* mobility	*kinD, mhqR, motA, motB*
IIG-Bs-27-28	*ymzD-ymaC;* protease	*ymzD, ymzC, pksA, pksB, pksC, pksD, pksE, acpK, pksF, pksG, pksH, pksI, pksJ, pksL, pksM, pksN, pksR, pksS, ymzB, ymaE, aprX, ymzE, ymaC, ymaD, ebrB, ebrA, ymaG*
IIG-Bs-27-29	*yulE-yugM;* chemotaxis	*yulE, yulD, yulC, yulB, yuxG, tlpB, mcpA, tlpA, mcpB, tgl, yuzH, yugU, yugT, yugS, yugP, yuzI, mstX, yugO, yugN, yugM*
IIG-Bs-27-30	*yefB-yetO; rapH* regulatory gene for differentiation, spore coat protein, rhamnosgalacturonan degradation	*yefB, yefC, yeeA, yeeB, yeeC, yeeD, yezA, yezG, yeeF, yeeG, rapH, phrH, yeeI, yeeK, yezE, yesE, yesF, cotJA, cotJB, cotJC, yesJ, yesK, yesL, yesM, yesN, yesO, yesP, yesQ, yesR, yesS, rhgT, yesU, yesV, yesW, yesX, yesY, yesZ, yetA, lplA, lplB, lplC, lplD, yetF, yetG, yetH, yetI, yezD, yetJ, yetK, yetL, yetM, yetN, yetO*
IIG-Bs-27-31	*yhfM-yhfQ;* protease	*yhfM, yhfN, aprE, yhfO, yhfP, yhfQ*
IIG-Bs-27-33	*sspH-yfiV*; maltose utilisation, spore coat protein, esterase	*sspH, yfjF, yfjE, yfjD, yfjC, yfjB, yfjA, malA, malR, malP, malQ, yfiC, catD, catE, yfiF, yfiG, yfiH, yfiI, yfiJ, yfiK, yfiL, yfiM, yfiN, padR, estB, yfiQ, yfiR, yfiS, yfiT, yfiU, yfiV*
IIG-Bs-27-35	*comQ-comA;* quorum sensing competence regulator	*comQ, comX, comP, comA*
IIG-Bs-27-36	*yuaB;* biofilm	*yuaB*
IIG-Bs-27-37	*ywrJ-ywqG;* spore coat protein	*ywrJ, cotB, cotH, cotG, ywrF, ywrE, ywrD, ywrC, ywrB, ywrA, ywqO, ywqN, ywqM, ywqL, ywqK, ywqJ, ywqI, ywqH, ywqG*
IIG-Bs-27-38	*yvzF-gerAC*; germination	*yvzF, gerAA, gerAB, gerAC*
IIG-Bs-27-39	*yojG-yodJ;* germination	*yojG, yojF, yoyC, yojE, gerT, yojB, yojA, yodA, yodB, yodC, yodD, yodE, yoyD, yodF, ctpA, yodH, yodI, yodJ*
IIG-Bs-27-40	*yueI-yukJ*	*yueI, yueH, yueG, yueF, yuzE, yuzF, yueE, yueD, yueC, yueB, yukB, yukC, yukD, yukE, yukF, ald, yukJ*
IIG-Bs-27-41	*yttB-ytoA*	*yttB, yttA, bceB, bceA, bceS, bceR, ytrF, ytrE, ytrD, ytrC, ytrB, ytrA, ytzC, ytqA, ytqB, ytpB, ytpA, ytoA*
IIG-Bs-27-42	*yoxC-yobB;* pectine esterase	*yoxC, yoxB, yoaA, yoaB, yoaC, yoaD, yoaE, yoaF, yoaG, yozQ, yoaH, yoaI, exlX, yoaK, pelB, yoaM, yozS, yoaN, yoaO, yoaP, yoaQ,*

		yozT, yozF, yoaR, yoaS, yozG, yoaT, yoaU, cyeA, yoaW, yoaZ, penP, yobA, yozU, yobB
IIG-Bs-27-43	*manA-yjdJ*	*manP, manA, yjdF, yjdG, yjdH, yjdI, yjzH, yjdJ*
IIG-Bs-27-44	*yxkM-pepT;* peptidase, galactose utilization	*yxkH, msmX, yxkF, aldY, yxkD, yxkC, galE, yxkA, yxjO, yxjN, yxjM, yxjL, pepT*
IIG-Bs-27-45	*yxaM-yxaB*	*yxaM, yxaL, yxaJ, yxaI, yxaH, qodI, yxaF, yxnA, yxaD, yxzK, yxaC, yxaB*
IIG-Bs-27-46	*bglS-yxiF;* β-glucosidase	*bglS, licT, yxiP, yxiO, deaD, yxiM, yxzI, yxzJ, yxiK, yxiJ, yxiI, yxzG, yxiH, yxiG, yxzC, yxiF*
IIG-Bs-27-47	*yncM-fosB;* TAT	*yncM, ynzK, cotC, tatAC, yndA, yndB, ynzB, yndD, yndE, yndF, yndG, yndH, yndJ, yndK, yndL, yndM, fosB*
IIG-Bs-27-47-1	*pgcM-crh;* maltooligosaccharide utilisation	*pgcM, malL, malK, yvdJ, mdxG, mdxF, mdxE, mdxD, mdxR, yvdD, yvdC, yvdB, yvdA, yvcT, yvcS, yvcR, yvcQ, yvcP, yvcN, crh*
IIG-Bs-27-47-2	*ypsA-sspM*	*ypsA, cotD, yprB, yprA, ypqE, ypqA, yppG, yppF, yppE, yppD, sspM*
IIG-Bs-27-47-3	*araE-yvfH;* arabinose transport, arabinogalactan	*araE, araR, yvbT, yvbU, cyeB, yvbW, RNA_56, yvbX, yvbY, yvfW, yvfV, yvfU, yvfT, yvfS, yvfR, rsbQ, rbsP, ganB, ganA, ganQ, ganP, cycB, ganR, yvfI, yvfH*
IIG-Bs-27-47-4	*yraN-levR;* chitosanase, alcohol dehydrogenase, levan degradation	*yraN, yraM, csn, yraL, yraK, yraJ, yraI, yraH, yraG, yraF, adhB, yraE, yraD, yraB, yrzP, adhA, yraA, sacC, levG, levF, levE, levD, levR*
IIG-Bs-27-47-5	*yweA-ywdH;* spore coat glycosylation	*yweA, spsL, spsK, spsJ, spsG, spsF, spsE, spsD, spsC, spsB, spsA, ywdL, ywdK, ywdJ, ywdI, ywdH*
IIG-Bs-27-47-6	*nagA-yvzB*; N-acetylglucosamin degradation	*nagA, nagBA, yvoA, yvnB, yvnA, cypX, yvmC, yvmB, yvmA, yvlD, yvlC, yvlB, yvlA, yvkN, yvzB*
IIG-Bs-27-47-8	*sspI-ysdB;* arabinan degradation, glycolat oxidation	*sspI, ysfB, glcD, glcF, ysfE, cstA, abfA, araQ, araP, araN, araM, araL, araD, araB, araA, abnA, ysdC, ysdB*
IIG-Bs-27-47-9	*alkA-ybeC*	*alkA, adaAB, ndhF, ybcC, ybcF, ybcH, ybcI, ybzH, ybcLM, skfA, skfB, skfC, skfE, skfF, skfG, skfH, ybdG, ybdJ, ybdK, ybzI, ybdM, ybdN, ybdO, ybxG, csgA, ybxH, ybxI, cybC, ybyB, ybeC*
IIG-Bs-27-47-10	*yfhH-yfhP*	*yfhH, yfhI, sspK, yfhJ, yfhK, yfhL, yfhM, csbB, yfhO, yfhP*
IIG-Bs-27-47-11	*lytD-ywsA;* autolysin, germination, poly-γ-glutamate	*lytD, yvyI, gerBA, gerBB, gerBC, ywtG, ywtF, ywtE, pgdS, pgsE, pgsA, pgsC, pgsB, rbsR, rbsK, rbsD, rbsA, rbsC, rbsB, ywsB, ywsA*
IIG-Bs-27-47-12	*ykzT-ykuL*; 2,-4-dienoyl-CoA-reductase, murein transglycosylase	*ykzT, cheV, ykyB, ykuC, ykuD, ykuE, fadH, fadG, ykzU, ykuH, ykuI, ykuJ, ykuK, ykzF, ykuL*
IIG-Bs-27-47-13	*ycbC-ycbJ;* glucarate/galactarate degradation	*ycbC, ycbD, gudP, gudD, ycbG, garD, ycbJ*
IIG-Bs-27-47-14	*cotT-yjlB*	*cotT, yjeA, yjfA, yjfB, yjfC, yjgA, yjgB, yjgC, yjgD, yjhA, yjhB, yjiA, yjiB, yjiC, yjzI, yjjA, yjkA, yjkB, yjlA, yjlB*
IIG-Bs-27-47-15	*xynP-ynzJ;* xylan/xylose degradation	*xynP, xynB, xylR, xylA, xylB, yncB, yncC, alrB, yncE, yncF, cotU, ynzJ*
IIG-Bs-27-47-16	*ykzB-ykoT*	*ykzB, ykoL, ykoM, ykoN, ykoP, ykoQ, ykoS, ykoT*
IIG-Bs-27-47-17	*uxaC-spoIISA*	*uxaC, exuM, yjmC, yjmD, uxuA, uxuB, exuT, exuR, uxaB, uxaA, yjnA, yjoA, yjoB, rapA, phrA, xlyB, yjqA, yjqB, yjqC, xkdA, xre, yjzJ, xkdB, xkdC, xkzK, xkdD, xtrA, xpf, xtmA, xtmB, xkdE, xkdF, xkdG, xkzL, xkdH, xkdI, xkdJ, xkzM, xkdK, xkdM, xkdN, xkzB, xkdO, xkdP, xkdQ, xkdR, xkdS, xkdT, xkdU, xkzA, xkdV, xkdW, xkdX, xepA, xhlA, xhlB, xlyA, spoIISB, spoIISA*
IIG-Bs-27-47-18	*ykvN-ykvU*	*ykvN, ykvO, ykvP, ykzQ, ykvQ, ykzR, ykvR, ykvS, ykzS, ykvT, ykvU*

IIG-Bs-27-47-19	*yocH-yozC*	*yocH, yocI, misc_RNA32 (bsrB, 6S-2RNA), yocJ, yocK, yocK, yocL, yoyB, yocM, yozN, yocN, yozO, yozC*
IIG-Bs-27-47-20	*ypwA-ypzG*	*ypwA, kdgT, kdgA, kdgK, kdgR, kduI, kduD, ypvA, yptA, ypzG*
IIG-Bs-27-47-21	*yoyE-ypmR*	*yoyE, yodL, yodM, yozD, yoyF, yodN, yozE, yokU, kamA, yodP, yodQ, yodR, yodS, yodT, yoyG, cgeE, cgeD, cgeC, cgeA, cgeB, ypqP, msrB, msrA, ypoP, dinF, ypmT, ypmS, ypmR*
IIG-Bs-27-47-22	*padC-cotR*	*padC, yveG, yveF, racX, pbpE, sacB, levB, yveA, yvdT, yvdS, yvdR. yvdQ, yvdP, cotR*
IIG-Bs-27-47-23	Insertion *slrR* at *aprE* locus	
IIG-Bs-27-47-24	*pspA-yebAP*	*pspA, ydjG, ydjH, ydjI, ydjJ, iolT, bdhA, ydjM, ydjN, ydzJ, ydjO, ydjP, yeaA, cotA, gabP, ydzX, yeaB, yeaC, yeaD, yebA*
